# Spatial predictors of immunotherapy response in triple-negative breast cancer

**DOI:** 10.1038/s41586-023-06498-3

**Published:** 2023-09-06

**Authors:** Xiao Qian Wang, Esther Danenberg, Chiun-Sheng Huang, Daniel Egle, Maurizio Callari, Begoña Bermejo, Matteo Dugo, Claudio Zamagni, Marc Thill, Anton Anton, Stefania Zambelli, Stefania Russo, Eva Maria Ciruelos, Richard Greil, Balázs Győrffy, Vladimir Semiglazov, Marco Colleoni, Catherine M. Kelly, Gabriella Mariani, Lucia Del Mastro, Olivia Biasi, Robert S. Seitz, Pinuccia Valagussa, Giuseppe Viale, Luca Gianni, Giampaolo Bianchini, H. Raza Ali

**Affiliations:** 1 CRUK Cambridge Institute, University of Cambridge, Cambridge, UK; 2 National Taiwan University Hospital, College of Medicine, National Taiwan University and Taiwan Breast Cancer Consortium, Taipei, Taiwan; 3 Department of Gynecology, Brust Gesundheit Zentrum Tirol, Medical University Innsbruck, Innsbruck, Austria; 4 Fondazione Michelangelo, Milan, Italy; 5 Medical Oncology, Hospital Clínico Universitario de Valencia, Biomedical Research Institute INCLIVA, Valencia, Spain; 6 Medicine Department, Universidad de Valencia, Valencia, Spain; 7 Oncology Biomedical Research National Network (CIBERONC-ISCIII), Madrid, Spain; 8 San Raffaele Hospital, Milan, Italy; 9 IRCCS Azienda Ospedaliero-universitaria di Bologna, Bologna, Italy; 10 Department of Gynecology and Gynecological Oncology, Agaplesion Markus Krankenhaus, Frankfurt am Main, Germany; 11 Hospital Universitario Miguel Servet, Zaragoza, Spain; 12 Department of Oncology, Azienda Sanitaria Universitaria Friuli Centrale, Udine, Italy; 13 Hospital Universitario 12 de Octubre, Madrid, Spain; 14 3rd Medical Department, Paracelsus Medical University Salzburg, Salzburg, Austria; 15 Salzburg Cancer Research Institute-CCCIT, Salzburg, Austria; 16 Cancer Cluster Salzburg, Salzburg, Austria; 17 Department of Bioinformatics, Semmelweis University, Budapest, Hungary; 18 Cancer Biomarker Research Group, Research Centre for Natural Sciences, Institute of Enzymology, Budapest, Hungary; 19 NN Petrov Research Institute of Oncology, St. Petersburg, Russia; 20 IEO, Istituto Europeo di Oncologia, IRCCS, Milan, Italy; 21 Mater Private Hospital, Dublin and Cancer Trials Ireland Breast Group, Dublin, Ireland; 22 Fondazione IRCSS - Istituto Nazionale Tumori, Milan, Italy; 23 IRCCS Ospedale Policlinico San Martino, UO Clinica di Oncologia Medica, Genoa, Italy; 24 Dipartimento di Medicina Interna e Specialità Mediche (Di.M.I.), Università di Genova, Genoa, Italy; 25 Oncocyte Corporation, Irvine, CA USA; 26 University of Milan, Milan, Italy; 27 Department of Histopathology, Addenbrookes Hospital, Cambridge, UK

**Keywords:** Breast cancer, Tumour biomarkers, Immunotherapy

## Abstract

Immune checkpoint blockade (ICB) benefits some patients with triple-negative breast cancer, but what distinguishes responders from non-responders is unclear^1^. Because ICB targets cell–cell interactions^2^, we investigated the impact of multicellular spatial organization on response, and explored how ICB remodels the tumour microenvironment. We show that cell phenotype, activation state and spatial location are intimately linked, influence ICB effect and differ in sensitive versus resistant tumours early on-treatment. We used imaging mass cytometry^3^ to profile the in situ expression of 43 proteins in tumours from patients in a randomized trial of neoadjuvant ICB, sampled at three timepoints (baseline, *n* = 243; early on-treatment, *n* = 207; post-treatment, *n* = 210). Multivariate modelling showed that the fractions of proliferating CD8^+^TCF1^+^T cells and MHCII^+^ cancer cells were dominant predictors of response, followed by cancer–immune interactions with B cells and granzyme B^+^ T cells. On-treatment, responsive tumours contained abundant granzyme B^+^ T cells, whereas resistant tumours were characterized by CD15^+^ cancer cells. Response was best predicted by combining tissue features before and on-treatment, pointing to a role for early biopsies in guiding adaptive therapy. Our findings show that multicellular spatial organization is a major determinant of ICB effect and suggest that its systematic enumeration in situ could help realize precision immuno-oncology.

## Abstract (web summary)

Imaging mass cytometry is used to map the multicellular dynamics of immune checkpoint blockade-treated triple-negative breast cancer, finding that key proliferative fractions and cell–cell interactions drive response, and immunotherapy distinctively remodels tumour structure.

## Main

Immunotherapy has transformed the treatment of solid tumours but its best use in breast cancer remains unclear^4^. Triple-negative breast cancer (TNBC), which lacks hormone receptor and human epidermal growth factor 2 (HER2) expression, is an aggressive subtype for which new therapies are needed^5,6^. In TNBC, trials of immune checkpoint blockade (ICB) targeting the interaction between programmed death protein 1 (PD-1) and programmed cell death ligand 1 (PD-L1) have shown that some patients benefit^1,4,7^, but we lack a reliable biomarker to identify responders^1^

Interactions between proximate specialized cells in distinct activation states underpin the effect of ICB^2^. In cancer, chronic T cell stimulation leads to dysfunction, owing to interactions between cells expressing immune checkpoint receptors and ligands. ICB prevents this to invigorate dysfunctional T cells^2^ and these invigorated T cells must then interact with target cancer cells to induce cell death^8^. The efficacy of ICB therefore depends on both the cellular composition and multicellular organization of tumours because they orchestrate these interactions. Breast tumours are heterocellular ecosystems of cancer and tumour microenvironment (TME) cells^9–12^ that self-organize as distinct, recurring multicellular structures^13^. Despite this, the relationship between phenotypic spatial organization of tumours and ICB response has been little explored.

Although multicellular organization pre-treatment may indicate whether the immune response can be augmented by ICB, how ICB remodels tissue structure to achieve this remains obscure. Serial tumour sampling before, during and after treatment could uncover treatment-induced remodelling but is challenging in routine clinical practice. This may explain why the relationship between tissue dynamics during treatment and response is unknown.

To characterize the relationship between tissue structure, its dynamics on therapy and immunotherapy response in TNBC, we used imaging mass cytometry^3^ (IMC) to precisely quantify the phenotype, activation state and spatial location of cells in tumours sampled at three timepoints from patients enrolled in a randomized trial of neoadjuvant immunotherapy. We found that both the cellular composition and spatial organization of tumours pre-treatment were predictive of immunotherapy response, that sensitive tumours were distinct from resistant tumours early on-treatment and that response was best predicted by combining features from both timepoints.

## Longitudinal multiplexed imaging of TNBC

**Fig. 1:**
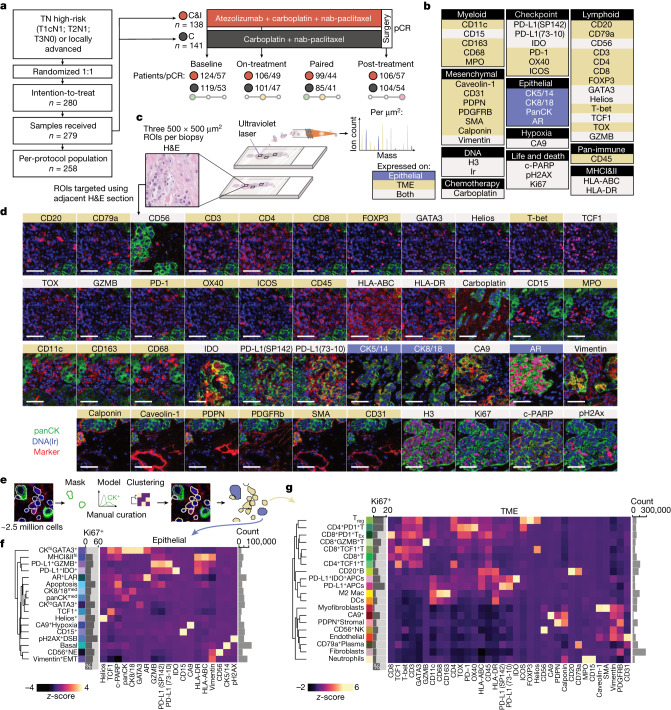
IMC workflow of the NeoTRIP immunotherapy trial. **a**, Flowchart of longitudinal tumour sampling from the NeoTRIP randomized clinical trial for high parameter imaging. **b**, Antibody panel targeting 43 protein markers expressed by epithelial (blue), TME (gold) or both (grey) cells. **c**, Schematic illustration of region of interest (ROI) selection for targeted multiplexed imaging by IMC, guided by an adjacent haematoxylin and eosin (H&E) section. **d**, Representative images of protein expression (cropped to fit; white scale bar, 50 µm). **e**, Semi-supervised workflow for distinguishing epithelial and TME cells from multiplexed images. **f**, Heatmap of median expression values for 17 epithelial cell phenotypes clustered using the proteins depicted on the *x* axis; right-sided grey bar chart depicts the number of cells per phenotype. **g**, As for **f**, for 20 TME cell phenotypes. C, chemotherapy; C&I, chemotherapy and immunotherapy; DC, dendritic cell; Mac, macrophage; NE, neuroendocrine; pCR, pathological complete response; TN, triple negative.

**Extended Data Fig. 1:**
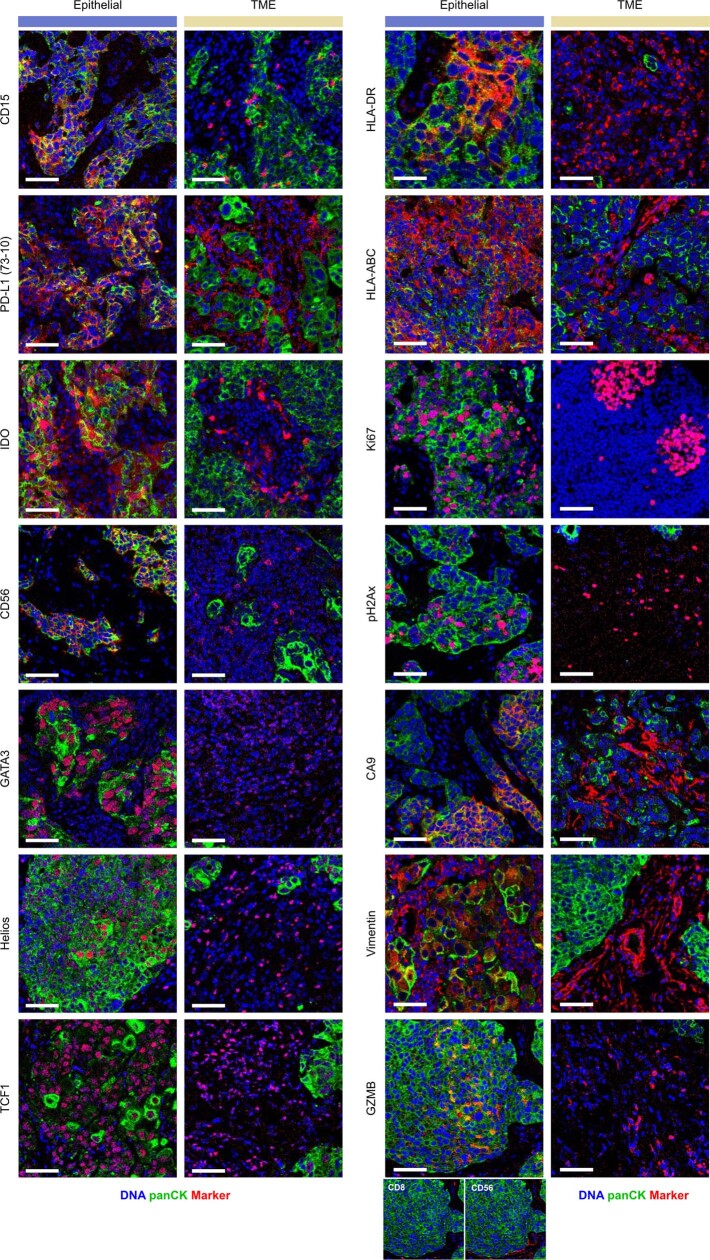
Localisation of proteins expressed by both epithelial and TME cells. White scale bars represent 50 µm.

**Extended Data Fig. 2:**
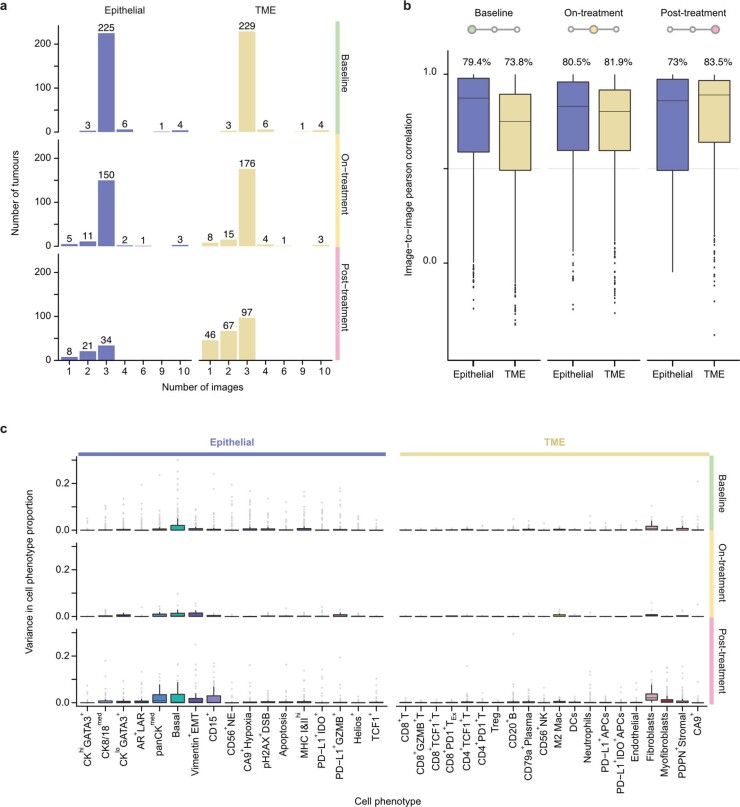
Analysis of intratumoural heterogeneity across ROIs. **a**, Bar plots showing the distribution of ROIs per tumour per timepoint separately for epithelial (blue) or TME cells (beige). **b**, Boxplots comparing the Pearson correlations of cell phenotype proportions for epithelial cell phenotypes (blue) and TME cell phenotypes (beige) between ROIs for each tumour. Values above whiskers are the percentage of correlations greater than 0.5. **c**, Boxplots showing variance of the proportion of each cell phenotype across ROIs for each tumour by timepoint. For all boxplots, boxes show 25th, 50th, and 75th centiles; whiskers indicate 75th centile plus 1.5x inter-quartile range and 25th centile less 1.5x inter-quartile range; points beyond whiskers are outliers.

**Extended Data Fig. 3:**
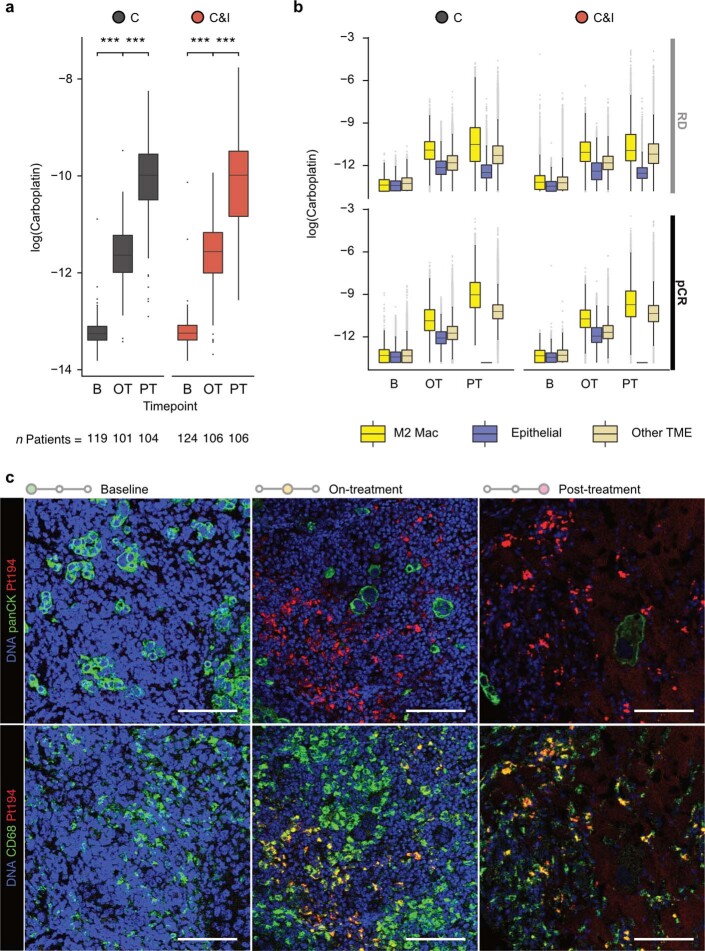
Detection of carboplatin in situ during therapy. **a**, Mean intracellular carboplatin detected per tumour by timepoint and arm. **b**, Amount of carboplatin detected in macrophages, epithelial cells and other TME cells by timepoint and arm. For both (**a**) and (**b**), Boxes show 25th, 50th, and 75th centiles; whiskers indicate 75th centile plus 1.5x inter-quartile range and 25th centile less 1.5x inter-quartile range; points beyond whiskers are outliers. ***P < 0.001 based on two-sided Wilcoxon tests. **c**, Representative images of carboplatin distribution in breast tumour tissue at baseline, on-treatment and post-treatment reveal co-localisation with CD68 indicating uptake by macrophages. White scale bars represent 100 µm. Baseline, B; On-treatment, OT; Post-treatment, PT; Chemotherapy, C; Chemotherapy and immunotherapy, C&I.

We used IMC to profile the expression of 43 proteins at subcellular resolution in tumour samples of formalin-fixed paraffin-embedded (FFPE) tissue collected from patients with TNBC enrolled in the NeoTRIP randomized controlled trial^14^ (Fig. 1 and Extended Data Figs. 1 and 2a). NeoTRIP was a trial of early TNBC that compared neoadjuvant chemotherapy (carboplatin and nab-paclitaxel) with chemotherapy plus anti-PD-L1 immunotherapy (carboplatin, nab-paclitaxel and atezolizumab) by 1:1 randomization^14^. We collected FFPE tissues at three timepoints for IMC (*n* = 279 patients): pre-treatment (baseline, *n* = 243), on the first day of the second treatment cycle (on-treatment, *n* = 207) and at surgical excision of the tumour bed following treatment (post-treatment, *n* = 210; Supplementary Tables 1–6). IMC uses laser ablation and time-of-flight mass spectrometry to detect antibodies conjugated to rare earth metal reporters to infer protein abundance at subcellular resolution^3^. We labelled tissues using a 43-plex IMC assay to precisely characterize the TME and key cancer cell phenotypes. In addition, we mapped carboplatin directly in situ by detecting platinum and found that levels in on-treatment and post-treatment samples were far greater than baseline, with much of the drug accumulating in macrophages (Extended Data Fig. 3). Our approach successfully generated 1,855 high-plex tissue images from both biopsies and excisions using FFPE samples collected prospectively as part of a randomized trial.

## Diverse cell phenotypes in TNBC

**Extended Data Fig. 4:**
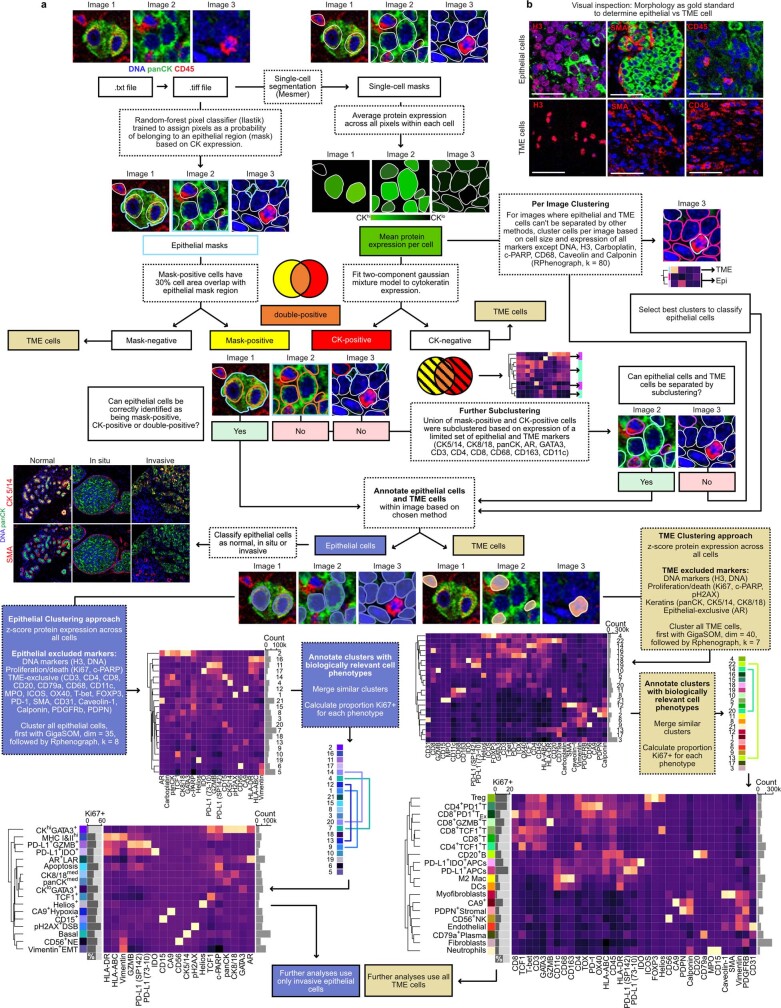
Flow chart of detailed cell phenotyping methodology. **a**, Workflow showing different approaches to characterising epithelial and TME cells as well as phenotyping the cells by compartment. Depicted cell segmentations are for illustration only. **b**, Curation of cells as epithelial or TME depends on visual inspection of cell morphology and staining patterns of specified markers. Cancer cells characterised by low cytokeratin and increased mesenchymal protein expression were frequent, and only reliably identified by multi-tiered and semi-supervised methods. White scale bars represent 50 µm.

**Extended Data Fig. 5:**
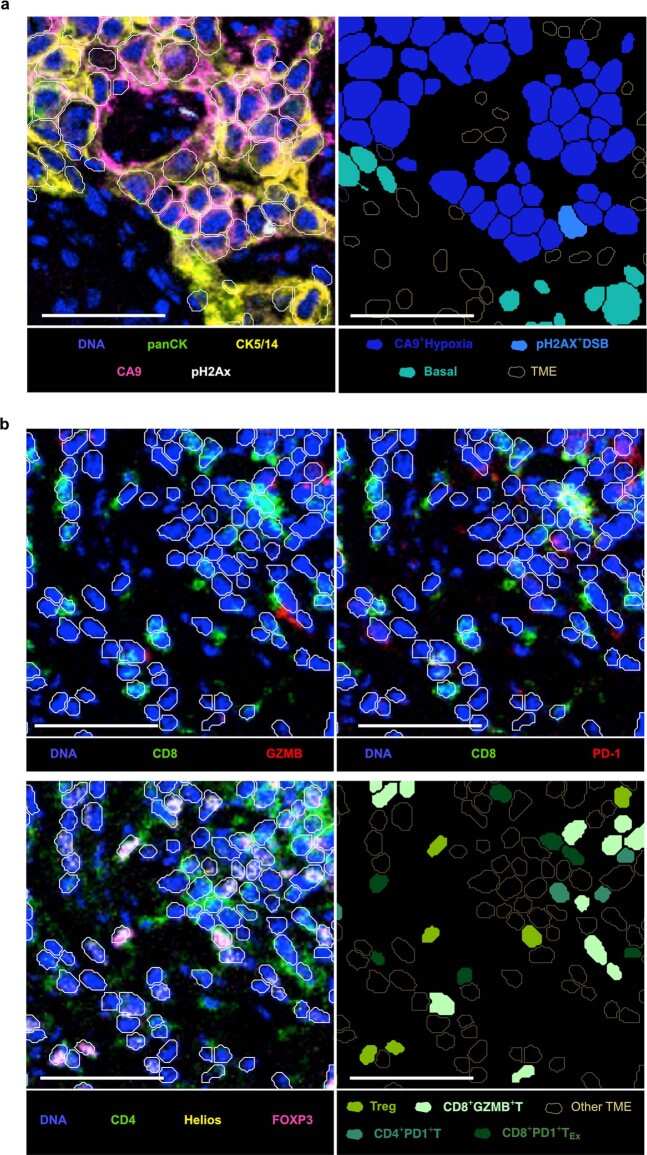
Illustrative examples of multiplexed images and corresponding cell phenotype assignments. **a**, Example of epithelial cell phenotypes and their protein expression **b**, Example of TME cell phenotypes and their protein expression. White scale bar is 50 µm.

To precisely characterize cell phenotypes in situ, we segmented single cells using deep learning^15^ and derived proteomic profiles. For cell phenotyping, we separated epithelial (cancer cells) and TME cells using several methods (Fig. 1e and Extended Data Fig. 4) and, taking cell morphology as the standard, selected the best performing. To discover salient cell phenotypes, we clustered single cells, limiting the proteins used for clustering to those relevant to epithelial or TME cells (Fig. 1f,g and Extended Data Fig. 5). Cell clustering resulted in 17 epithelial and 20 TME phenotypes (Supplementary Fig. 1). Epithelial cells were distinguished by markers of lineage, activation state and immunoregulation. Among TME cells, cytotoxic and helper T cells separated according to expression of PD-1 and TCF1 (encoded by *TCF7*; Extended Data Fig. 5b) which identifies stem-like T cells implicated in ICB response^16^. We also identified regulatory T (T_reg_) cells defined by FOXP3 expression and activated cytotoxic T cells with high granzyme B expression (CD8^+^GZMB^+^T). There were two cell phenotypes positive for PD-L1; these were both CD11c^+^ antigen presenting cells (APCs), of which one was IDO^+^. We also identified B cells, plasma cells, macrophages, dendritic cells, neutrophils, endothelial cells and three fibroblast phenotypes. We next correlated the cellular compositions of different images from the same tumour to estimate the extent of spatial heterogeneity (Extended Data Fig. 2b). Correlations between images from the same tumour varied between compartment and timepoint (from an average of 0.65 for TME cells at baseline, to 0.76 for TME cells post-treatment; Extended Data Fig. 2b). We also examined the variance per cell phenotype and found that it was generally low, with the highest being among basal epithelial cells (Extended Data Fig. 2c).

**Extended Data Fig. 6:**
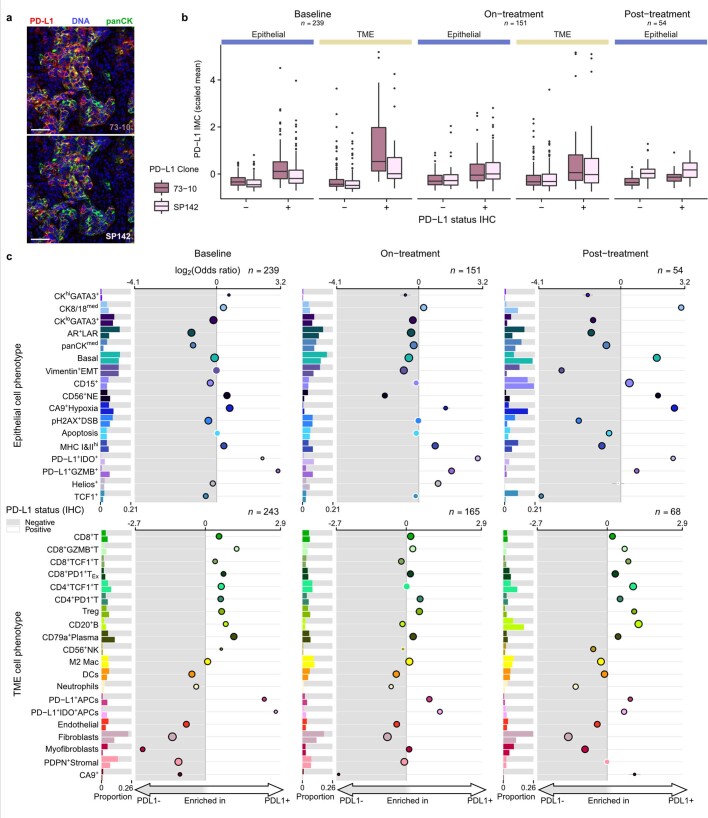
Associations between PD-L1 status and high dimensional imaging data. **a**, Representative images comparing the staining pattern of two different antibody clones targeting PD-L1. White scale bar represents 50µm. **b**, Boxplots of PD-L1 expression by IMC in tumour and TME cells separated by clinical PD-L1 status. Boxes show 25th, 50th, and 75th centiles; whiskers indicate 75th centile plus 1.5x inter-quartile range and 25th centile less 1.5x inter-quartile range; points beyond whiskers are outliers. **c**, Associations between cell phenotypes and clinical PD-L1 status depicted as log_2_ odds ratios derived from binomial generalised linear models where PD-L1 status was a predictor of cell phenotype proportion. Bar charts depict cell phenotype proportion relative to epithelial or TME cells, split by PD-L1 status. Immunohistochemistry, IHC.

**Extended Data Fig. 7:**
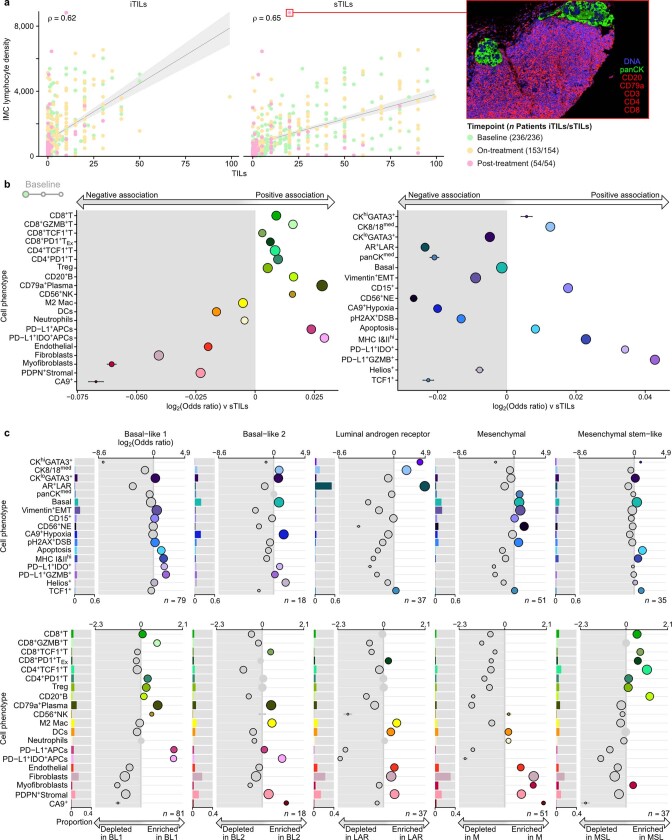
Associations between cell phenotypes, tumour infiltrating lymphocytes, and TNBC subtypes. **a**, Levels of lymphocyte density by IMC compared to intratumoural tumour infiltrating lymphocytes (iTILs) or stromal TILs (sTILs) per tumour per timepoint. ρ is Spearman rank correlation coefficient. An outlier tumour with high lymphocyte density post-treatment in IMC but low iTILs/sTILs is highlighted with a representative IMC image (lymphocyte lineage markers combined in red). **b**, Odds ratios from generalised linear models depicting associations between proportions of TME and epithelial cell phenotypes and levels of sTILs at baseline. Circles are point estimates and horizontal bars are 95% confidence intervals; circle size is inversely proportional to the standard error. **c**, Associations between cell phenotypes and TNBC subtypes depicted as log_2_ odds ratios where tumour subtype predicts cell phenotype proportion relative to all other subtypes. Number of tumours per subtype depicted in bottom right of each panel. Bar charts depict proportion of cell phenotype relative to all epithelial or TME cells within a given tumour subtype. Circles are point estimates and horizontal bars, where visible, are 95% confidence intervals; circle size is inversely proportional to the standard error. Basal-like 1, BL1; Basal-like 2, BL2; Luminal androgen receptor, LAR; Mesenchymal, M; Mesenchymal stem-like, MSL; Tumour infiltrating lymphocytes, TILs.

To determine the relevance of cell phenotypes to established tumour pathology, we investigated the relationship between cell phenotype proportions and relevant clinical features. In comparison with clinical PD-L1 status assessed by centralized pathology review, we confirmed that PD-L1 expression by IMC was greater in PD-L1-positive tumours (Extended Data Fig. 6a,b). Similarly, the cell phenotypes most enriched among PD-L1-positive tumours were characterized by the highest expression of PD-L1 (Extended Data Fig. 6c). All lymphoid cell phenotypes were also positively associated with PD-L1 status at baseline. Stromal infiltrating lymphocytes were also positively correlated with all lymphoid cells and PD-L1^+^ APCs (Extended Data Fig. 7a,b). We also investigated whether the abundance of cell phenotypes significantly differed between established transcriptomic subtypes of TNBC^17^ (Extended Data Fig. 7c). Both epithelial and TME cell phenotypes differed between tumour subtypes. The luminal androgen receptor subtype, for example, was characterized by the highest proportion of epithelial cells positive for androgen receptor (AR^+^LAR), and the mesenchymal tumour subtype contained the greatest proportion of all three stromal cell phenotypes. Together, these findings corroborated the quality of our multiplexed image data and cell phenotypes.

## Cancer–immune interactions predict ICB response

**Fig. 2:**
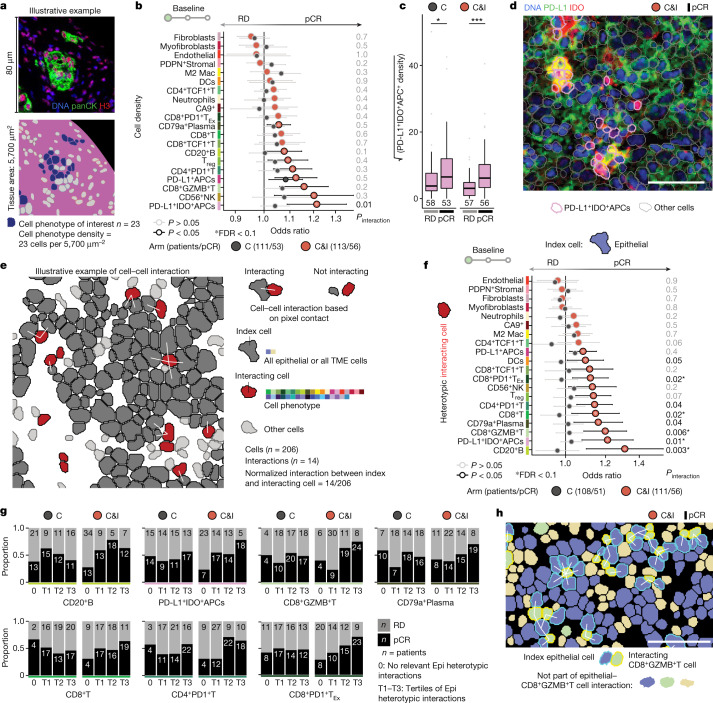
Spatial predictors of immunotherapy response at baseline. **a**, Schematic illustrating cell phenotype density calculation. **b**, Odds ratios for associations between cell density and pCR for TME cell phenotypes. **c**, Boxplot of PD-L1^+^IDO^+^APC density across treatment arms and response. Boxes show 25th, 50th and 75th centiles; whiskers indicate 75th centile plus 1.5 × inter-quartile range and 25th centile less 1.5 × inter-quartile range; points beyond whiskers are outliers. *P < 0.05, ***P < 0.001, based on two-sided Wilcoxon tests. **d**, Representative image of a tumour from a responder treated with immunotherapy with high PD-L1^+^IDO^+^APC density. **e**, Schematic illustrating principles of homotypic and heterotypic cell–cell interaction metrics. **f**, Odds ratios for associations between heterotypic epithelial-to-TME cell phenotypes and pCR. For **b** and **f**, Odds ratios are derived from univariate logistic regression: circles represent point estimates and whiskers indicate 95% confidence intervals. Depicted *P* values are derived from a term for interaction between the predictor and treatment in logistic regression models (including separate terms for the predictor and treatment). Asterisks indicate associations with an FDR < 0.1 by the Benjamini–Hochberg method. **g**, Bar charts of the proportion of tumours achieving pCR in patients with no selected epithelial–TME interactions per arm (0) or increasing tertiles of epithelial–TME interactions per arm (T1–T3). Numbers on bars are absolute numbers of patients in each category. **h**, Representative image of a tumour from a responder treated with immunotherapy with high baseline epithelial–CD8^+^GZMB^+^T cell interactions. All images were median filtered; white scale bar, 50 µm. RD, residual disease; Epi, epithelial.

**Extended Data Fig. 8:**
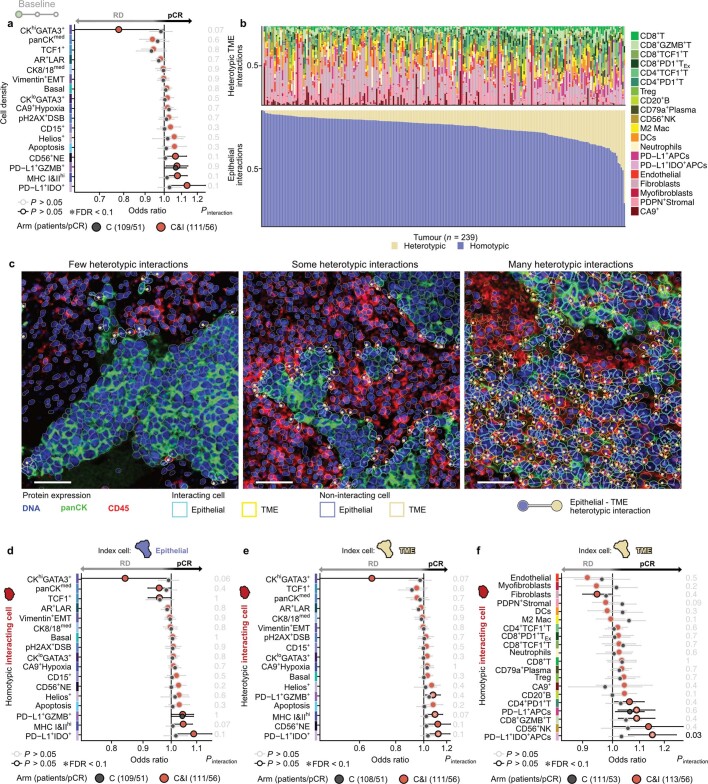
Baseline predictors of immunotherapy response. **a**, Odds ratios for associations between cell density and pCR for epithelial cell phenotypes. **b**, Proportion of TME cells interacting with epithelial cells and the proportion of heterotypic to homotypic interactions per tumour. **c**, Representative images of tumours with few, some, and many heterotypic interactions relative to total cell count. For all images, white scale bar represents 50 µm and a median filter was applied. **d**–**f**, Odds ratios for associations between cell-cell interactions and pCR among epithelial-to-epithelial cells (**d**), TME-to-epithelial cells (**e**) and TME-to-TME cells (**f**). For **a**, **d**–**f**, Odds ratios are derived from univariate logistic regression: circles represent point estimates and whiskers indicate 95% confidence intervals. Depicted *P*-values are derived from a term for interaction between the predictor and treatment in logistic regression models (including separate terms for the predictor and treatment) and have not been adjusted for multiple tests. Asterisks indicate *P*-values associated with false discovery rate <0.1 using the Benjamini-Hochberg method. False discovery rate, FDR; Pathological complete response, pCR; Residual disease, RD; Chemotherapy, C; Chemotherapy and immunotherapy, C&I.

We asked whether the tissue structure of treatment-naive tumours is a determinant of response to immunotherapy. An advantage of neoadjuvant trials such as NeoTRIP (in which primary treatment is given before surgical excision of the tumour) is that the surrogate end point of pathological complete response (pCR), defined as the absence of invasive cancer cells after treatment, can be used to identify responders before time-to-event follow-up has matured^7,14,18^. To evaluate the link between tissue structure and response, we fit logistic regression models to predict pCR separately by randomization arm and included a term for statistical interaction (*P*_interaction_) to test whether the association between a given feature and response significantly differed by treatment. To estimate the impact of multiple testing, we also computed the false discovery rate (FDR). We first asked whether the densities of different epithelial and TME cell phenotypes differed in their capacity to predict response (Fig. 2a,b and Extended Data Fig. 8a). Only PDL1^+^IDO^+^APC density predicted ICB (but not chemotherapy) response, although this was associated with an elevated FDR (*P*_interaction_ = 0.01, FDR = 0.3; Fig. 2b–d and Supplementary Tables 7 and 8).

**Extended Data Fig. 9:**
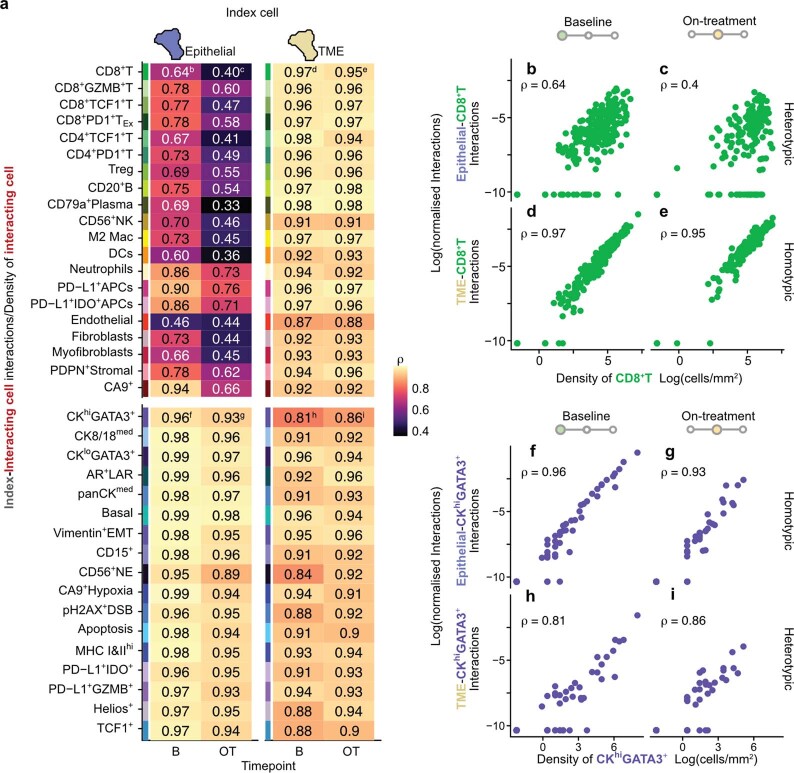
Correlations between cell phenotype density and cell-cell interactions. **a**, Heatmap depicting associations between density of a cell phenotype and interactions between that cell phenotype and all epithelial or TME cells as indicated by the index cell headings. Numbers are spearman rank correlation coefficients. **b**–**i**, Scatterplots illustrating selected relationships between density and cell-cell interactions indicated in (**a**). ρ denotes Spearman rank correlation coefficient. Zeros have been retained for each plot by adding a value 0.3x the smallest value per plot before taking the log value. Baseline, B; On-treatment, OT.

**Extended Data Fig. 10:**
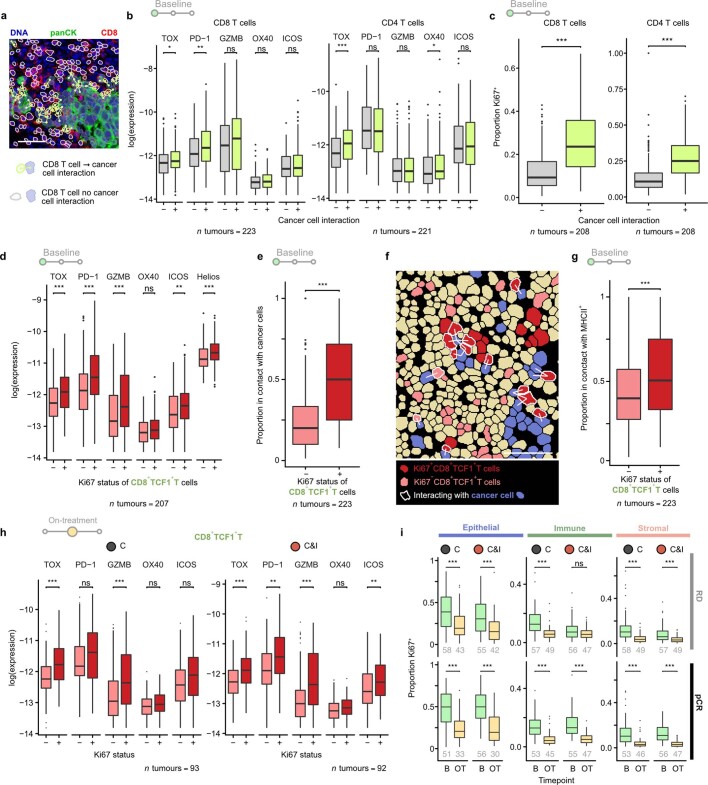
Differential T cell activation by cancer cell contact and proliferation status. **a**, Representative image of interactions between CD8 T cells and epithelial cells. **b**, Boxplots of mean expression levels (per tumour) of activation markers for T cells in contact or not in contact with cancer cells at baseline. **c**, Proportion of T cells positive for Ki67 in contact or not in contact with tumour cells at baseline. **d**, Boxplots of mean expression levels of activation markers in Ki67^+^ versus Ki67^−^ CD8^+^TCF1^+^T cells at baseline. **e**, Proportion of Ki67^+^ and Ki67^−^ CD8^+^TCF1^+^T cells in contact with cancer cells at baseline. **f**, An example of a baseline tumour with Ki67^+^ and Ki67^−^CD8^+^TCF1^+^T cells in contact with cancer cells. **g**, Proportion of Ki67^+^ and Ki67^−^ CD8^+^TCF1^+^T cells in contact with MHCII^+^ cancer cells at baseline. **h**, Boxplots of mean expression levels of activation markers in Ki67^+^ versus Ki67^−^ CD8^+^TCF1^+^T cells early on-treatment for each treatment arm. **i**, Proportion of Ki67^+^ epithelial, immune and stromal cells by timepoint, treatment arm and response. Note all values are the mean per tumour (i.e., each tumour contributes one data point per measurement). For all boxplots, boxes show 25th, 50th, and 75th centiles; whiskers indicate 75th centile plus 1.5x inter-quartile range and 25th centile less 1.5x inter-quartile range; points beyond whiskers are outliers. Asterisks denote *P*-values < 0.05 (*), < 0.01 (**) or < 0.001 (***) based on two-sided Wilcoxon tests. A median-filter was applied to **a** and, for all images, white scale bar is 50 µm. Baseline, B; On-treatment, OT; Chemotherapy, C; Chemotherapy and immunotherapy, C&I.

Because ICB modulates interactions among immune and cancer cells, we explored whether different cell–cell interactions were associated with response to immunotherapy but not chemotherapy. Cells were deemed to be interacting if they were in direct contact (Fig. 2e). For all epithelial cells, we computed the number of interactions with each epithelial cell phenotype (homotypic interactions) and with each TME cell phenotype (heterotypic interactions), normalizing by the total number of cells present (Fig. 2e). This approach gave greater weight to cells with multiple interactions. The distribution of cancer–TME interactions was a continuum across tumours (Extended Data Fig. 8b,c); hence, we modelled interactions as continuous predictors. Among epithelial–epithelial interactions four cell phenotypes were associated with outcome, but none of the estimates differed significantly between treatment arms (Extended Data Fig. 8d and Supplementary Tables 9 and 10). In stark contrast, associations between eight epithelial–TME interactions and response significantly differed between treatments, with epithelial–CD20^+^B (*P*_interaction_ = 0.003, FDR = 0.06) and epithelial–CD8^+^GZMB^+^T (*P*_interaction_ = 0.006, FDR = 0.06) cell interactions showing the greatest differential effect (Fig. 2f–h and Supplementary Tables 9 and 10). Repeating this analysis for TME cells did not, however, uncover significant predictors of differential response (Extended Data Fig. 8e,f and Supplementary Tables 9 and 10). Heterotypic epithelial interactions were only moderately correlated with corresponding cell densities, suggesting they reflect distinct aspects of tumour organization (Extended Data Fig. 9). Because activated T cell–cancer cell interactions predicted ICB response, we investigated whether T cells in contact with cancer cells were functionally distinct from other T cells (Extended Data Fig. 10a). We found that contact with a cancer cell was associated with higher expression of key activation markers (TOX and PD-1 for cytotoxic T cells; TOX and OX40 for T helper cells; Extended Data Fig. 10b), and that T cells contacting cancer cells were much more likely to be proliferating (Extended Data Fig. 10c). These findings corroborate the functional significance of cell–cell interactions.

In summary, cancer–immune interactions are functionally significant pre-existing markers of the potential for invigorating the intratumoural immune response by ICB.

## Proliferative fractions predict ICB response

**Fig. 3:**
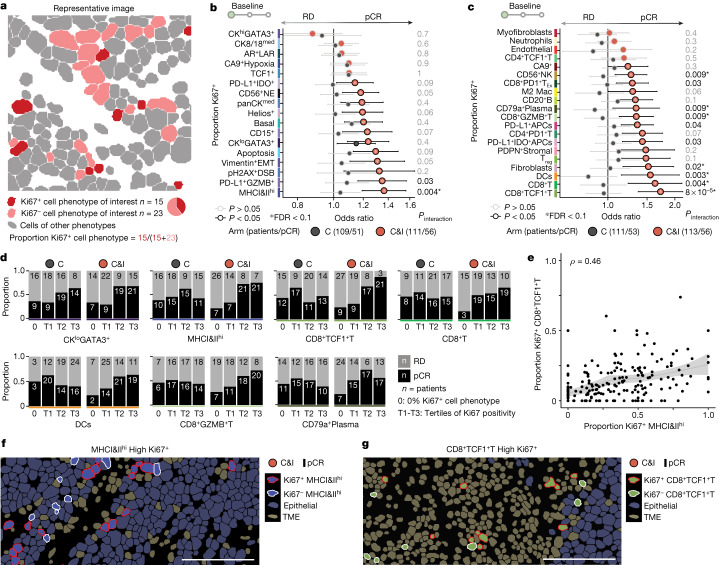
Proliferative fractions of cancer and TME cell phenotypes predict response to immunotherapy. **a**, Schematic illustrating calculation of cell phenotype-specific proliferative fractions (proportion of Ki67^+^ cells). **b**,**c**, Odds ratios for associations between proliferative fraction and pCR for epithelial (**b**) and TME (**c**) cell phenotypes. Odds ratios are derived from univariate logistic regression: circles represent point estimates and whiskers indicate 95% confidence intervals. Depicted *P* values are derived from a term for interaction between the predictor and treatment in logistic regression models (including separate terms for the predictor and treatment) and have not been adjusted for multiple tests. Asterisks indicate associations with an FDR < 0.1 by the Benjamini–Hochberg method. **d**, Bar charts of the proportion of tumours achieving pCR in patients with no Ki67^+^ cells of the selected phenotype per arm (0) or increasing proportion of Ki67^+^ cells per arm, as quantified by tertiles (T1–T3). Absolute numbers of patients in each category are depicted inside bar charts. **e**, Relationship between proliferative fraction of CD8^+^TCF1^+^T cells and MHCI&II^hi^ cells. *ρ* is Spearman rank correlation coefficient. Shaded area represents 95% confidence interval of the loess regression line. **f**,**g**, Representative images of tumours from immunotherapy-treated responders with high proliferative fractions of MHCI&II^hi^ cells (**f**) and CD8^+^TCF1^+^T cells (**g**). White scale bar, 100 µm.

Because immunotherapy induces T cell proliferation^16^, the fraction proliferating before treatment could modify its effect. Our finding that T cells in contact with cancer cells were more often Ki67^+^ also implicated proliferation in ICB response. We therefore computed the proportion of Ki67^+^ cells per phenotype (proliferative fraction) and tested for associations with pCR (Fig. 3a–c, Supplementary Table 11 and Supplementary Fig. 2). Strikingly, the proliferative fraction of just one cell phenotype was associated with response in the chemotherapy arm (CK^lo^GATA3^+^ epithelial cells), but when patients were treated with immunotherapy, 12 epithelial and 16 TME cell phenotypes predicted response (Fig. 3b,c and Supplementary Table 11). The proliferative fraction of MHCI&II^hi^ cells was the strongest predictor of immunotherapy response among epithelial (cancer) cells (*P*_interaction_ = 0.004, FDR = 0.04), whereas the proliferative fraction of CD8^+^TCF1^+^T cells was the strongest immunotherapy response predictor overall (*P*_interaction_ = 8 × 10^−5^, FDR = 0.003). These features (proliferative fractions of MHCI&II^hi^ cancer cells and CD8^+^TCF1^+^T cells) were, however, only moderately correlated (*ρ* = 0.46; Fig. 3e). Notably, CD8^+^TCF1^+^T cells are a stem-like population that underlies the proliferative burst induced by ICB^16^. Despite the proliferative fraction of CD8^+^TCF1^+^T cells being the strongest predictor, neither their overall density nor their interactions with TME cells were associated with response, underscoring that proliferative fractions enrich for cells in distinct activation states (Fig. 2f and Extended Data Fig. 8f). Indeed, proliferating CD8^+^TCF1^+^T cells showed significantly higher levels of all key activation markers (TOX, PD-1, GZMB, ICOS, Helios; Extended Data Fig. 10d) and were more often in contact with cancer cells (Extended Data Fig. 10e,f) and with MHCII^+^ cells, in keeping with past reports that these stem-like T cells reside in MHCII^+^ niches^19^ (Extended Data Fig. 10g). Pre-treatment proliferative fractions therefore enrich for cells in distinct activation states and identify phenotypes that predict ICB response.

## On-treatment ICB response predictors

**Fig. 4:**
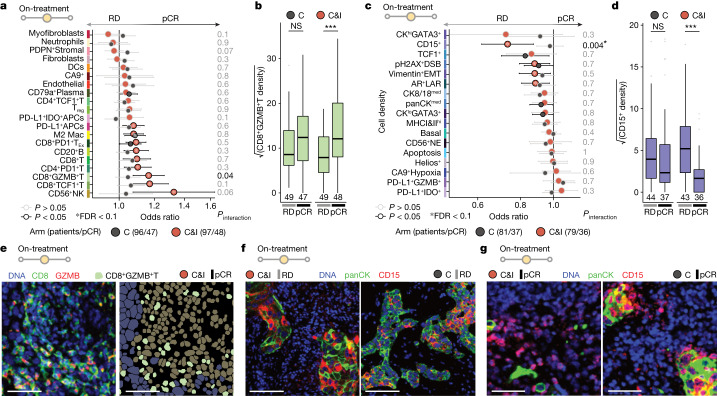
Cell phenotypes predictive of immunotherapy response early on-treatment. **a**, Odds ratios for associations between cell density and pCR for epithelial cell phenotypes. **b**, Boxplot of CD8^+^GZMB^+^T epithelial cell density across treatment arms and response. **c**, Odds ratios for associations between cell density and pCR for epithelial cell phenotypes. For **a** and **c**, odds ratios are derived from univariate logistic regression: circles represent point estimates and whiskers indicate 95% confidence intervals. Depicted *P* values are derived from a term for interaction between the predictor and treatment in logistic regression models (including separate terms for the predictor and treatment) and have not been adjusted for multiple tests. Asterisks indicate associations with an FDR < 0.1 by the Benjamini–Hochberg method. **d**, Boxplot of CD15^+^ cell density across treatment arms and response. For **b** and **d**, boxes show 25th, 50th and 75th centiles; whiskers indicate 75th centile plus 1.5 × inter-quartile range and 25th centile less 1.5 × inter-quartile range; points beyond whiskers are outliers. ***P < 0.001, based on two-sided Wilcoxon tests. **e**, Representative image of a tumour from an immunotherapy-treated responder with high on-treatment CD8^+^GZMB^+^T cell density. On the left are the imaging data and on the right are the cell phenotype masks. White scale bar, 50 µm. **f**, Representative images of CD15 mosaic expression pattern on-treatment in two tumours. White scale bar, 100 µm. **g**, Representative image of CD15^+^ tumours with CD15^+^ TME cells nearby. White scale bar, 50 µm. All images are median filtered. NS, not significant.

**Extended Data Fig. 11:**
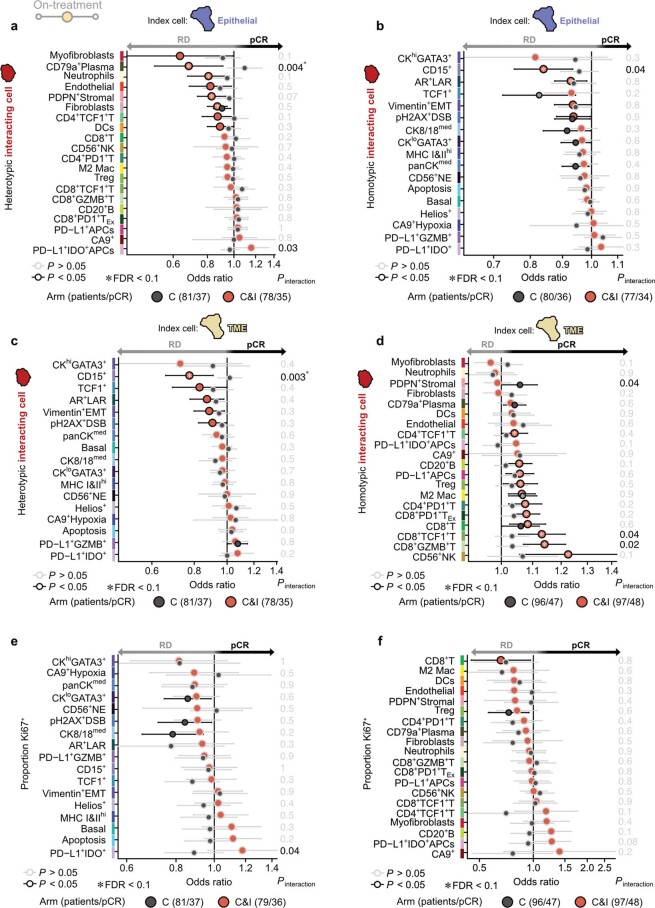
Early on-treatment predictors of immunotherapy response. **a** - **d**, Odds ratios for associations between cell-cell interactions and pCR among epithelial-to-TME (**a**), epithelial-to-epithelial (**b**), TME-to-epithelial (**c**) and TME-to-TME (**d**) cells. **e**, **f**, Odds ratios for associations between proliferative fraction and pCR for Epithelial (**e**) and TME (**f**) cell phenotypes. Odds ratios are derived from univariate logistic regression: circles represent point estimates and whiskers indicate 95% confidence intervals. Depicted *P*-values are derived from a term for interaction between the predictor and treatment in logistic regression models (including separate terms for the predictor and treatment) and have not been adjusted for multiple tests. Asterisks indicate *P*-values associated with false discovery rate <0.1 using the Benjamini-Hochberg method. False discovery rate, FDR; Tumour microenvironment, TME; Pathological complete response, pCR; Residual disease, RD; Chemotherapy, C; Chemotherapy and immunotherapy, C&I.

Cell state and context before therapy reflect pre-existing intercellular dynamics, but how they are modified early on-treatment may also reveal whether, should a treatment course be completed, a tumour will ultimately respond. Using biopsies taken early on-treatment (first day of second treatment cycle), we investigated the link between on-treatment cell densities, cell–cell interactions and immunotherapy response (Fig. 4a–d, Extended Data Fig. 11a–d and Supplementary Tables 7–11). The correlation structure of cell densities and their corresponding cell–cell interaction metrics echoed that pre-treatment: heterotypic epithelial (cancer–TME) interactions were moderately correlated with cell densities whereas other cell–cell interaction metrics were highly correlated (Extended Data Fig. 9). Among heterotypic epithelial interactions, only epithelia interactions with CD79a^+^Plasma cells were significantly enriched in tumours resistant to immunotherapy but not chemotherapy (*P*_interaction_ = 0.004, FDR = 0.09); the density of CD79a^+^Plasma cells was not, however, associated with response (Fig. 4a and Extended Data Fig. 11a). Among other TME cell densities, only CD8^+^GZMB^+^T cells showed significant differential immunotherapy response prediction (*P*_interaction_ = 0.04, FDR = 0.5) and, consistent with their strong correlation, this also held for homotypic CD8^+^GZMB^+^T interactions (*P*_interaction_ = 0.02, FDR = 0.3) (Fig. 4a,b,e and Extended Data Fig. 11d), but with elevated FDRs. The CD15^+^ epithelial (cancer) cell phenotype was distinct because it was associated with resistance to immunotherapy (but not chemotherapy) when quantified as a density (*P*_interaction_ = 0.004, FDR = 0.08) or cell–cell interaction metric (heterotypic *P*_interaction_ = 0.003, FDR = 0.05; homotypic *P*_interaction_ = 0.04, FDR = 0.8; Fig. 4c,d and Extended Data Fig. 11b). In some ICB-resistant cases, expression of CD15 by cancer cells was characterized by a striking mosaic expression pattern for which clear CD15^+^CK^lo^ cells were admixed with CD15^−^CK^hi^ cells, suggestive of discrete phenotypic state transitions (Fig. 4f). We also observed foci of CD15^+^ cancer cells surrounded by CD15^+^ leukocytes, implicating heterotypic interactions as possible drivers of state transition (Fig. 4g).

Given the predictive value of proliferation in treatment-naive tumours, we asked whether it would perform similarly on-treatment. We found that although the functional significance of proliferation was largely preserved on-treatment, proliferation itself was reduced and proliferating cell fractions were not predictive of response (Extended Data Figs. 10h,i and 11e,f).

In conclusion, markers of outcome on-treatment were distinct from those in treatment-naive tumours. Response to immunotherapy was characterized by accumulation of CD8^+^GZMB^+^T cells, whereas heterotypic CD79a^+^Plasma cell interactions and CD15^+^ cancer cells marked resistant tumours.

## ICB-induced cellular dynamics

**Fig. 5:**
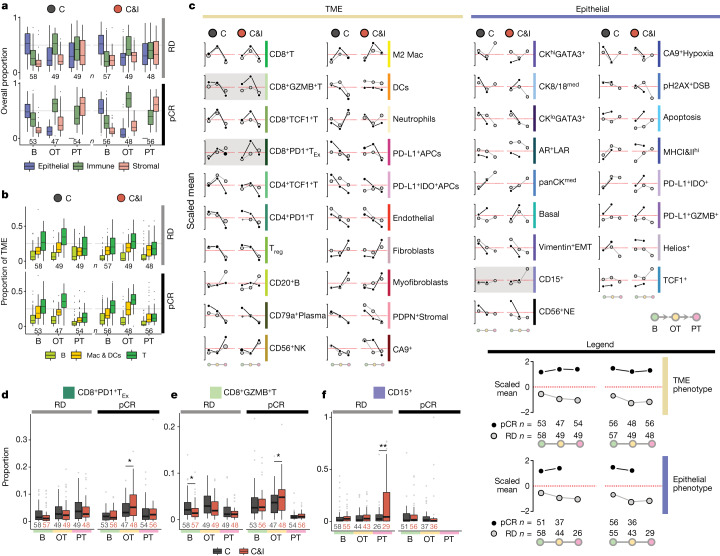
Dynamics of immunotherapy response. **a**, Boxplots depicting distributions of epithelial, immune and stromal cell proportions per patient separated by timepoint, treatment arm and response. (Spurious epithelial cell detections in the post-treatment arm among responders were removed.) **b**, Boxplots depicting proportion distributions per patient of key leukocytes: B (CD20^+^B, CD79a^+^Plasma), Macs & DCs (M2 Mac, DCs, PD-L1^+^ APCs, PD-L1^+^IDO^+^ APCs) and T (all T cell phenotypes, including T_reg_ cells) cells, relative to all TME cells, separated by timepoint, treatment arm and response. For **a** and **b**, *n* denotes number of patients. **c**, Trends of TME cell composition enriched or depleted across timepoint, treatment arm and response, depicted as a line plot with scaled mean (*Z*-scores) derived from proportions of each cell phenotype within their compartment. Circle sizes are inversely proportional to scaled variance. Numbers of patients per timepoint are indicated in the legend. Cell phenotypes with the most distinct differences between dynamics of treatment arm and response are shaded and represented as boxplots in **d**, **e** and **f**. **d**–**f**, Boxplots depicting CD8^+^PD1^+^T_Ex_ cells (**d**), CD8^+^GZMB^+^T cells (**e**) and CD15^+^ cells (**f**) as a proportion of all TME cells (**d**,**e**) or all epithelial cells (**f**) per patient across treatment arms, response and timepoints. Cell phenotypes with the largest differences in dynamics between treatment arms are depicted. *P < 0.05, **P < 0.01, based on two-sided Wilcoxon tests. For all boxplots (**a**, **b**, **d**, **e** and **f**), boxes show 25th, 50th and 75th centiles; whiskers indicate 75th centile plus 1.5 × inter-quartile range and 25th centile less 1.5 × inter-quartile range; points beyond whiskers are outliers. B, baseline; OT, on-treatment; PT, post-treatment.

Finding distinct drivers of immunotherapy response on-treatment compared with baseline led us to investigate the cellular dynamics of neoadjuvant immunotherapy. For an overall survey of tissue composition, we aggregated cell phenotypes into three main categories (epithelial: all cancer cells; stromal: fibroblasts, PDPN^+^Stromal, endothelial; and immune: all remaining TME phenotypes), and this revealed conserved dynamics across subgroups. Immune cells increased dramatically on-treatment and decreased after treatment, accompanied by a reduction in epithelial (cancer) cells and increase in stromal cells (Fig. 5a). This pattern was repeated across all groups, but the degree of change differed: the early increase in immune cell fraction was greater among responders, and greatest among responders treated with immunotherapy. We explored the dynamics of key leukocytes (T cells, B cells and APCs, namely macrophages and dendritic cells) relative to all TME cells to understand which drove the on-treatment immune response (Fig. 5b). T cells were the most abundant cell type and showed a characteristic increase on-treatment followed by a fall after treatment. APCs composed a lesser proportion of the TME, but their dynamics mirrored that of T cells. The on-treatment increase in T cells and APCs was also most pronounced among responders treated with immunotherapy. B cells, by contrast, occupied a similar proportion of the TME at all timepoints with only a modest decrease in responders.

**Extended Data Fig. 12:**
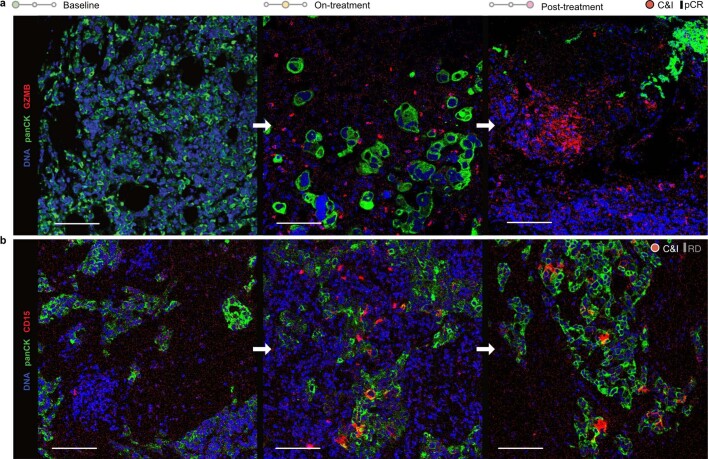
Representative images of a tumour across timepoints. **a**, Representative image of a tumour from a patient who responded to immunotherapy treatment illustrating changes in GZMB^+^ cells. **b**, Representative image of a tumour from a patient with residual disease following neoadjuvant immunotherapy treatment illustrating changes in CD15^+^ cells over time. White scale bar is 100 µm. Pathological complete response, pCR; Residual disease, RD; Chemotherapy, C; Chemotherapy and immunotherapy, C&I.

To identify intratumoural changes characteristic of ICB, we compared the mean proportions of all cell phenotypes over time (Fig. 5c). Response to ICB was characterized by greater infiltration on-treatment of CD8^+^GZMB^+^T and CD8^+^PD1^+^T_Ex_ cells (Fig. 5d,e and Extended Data Fig. 12a). Among non-responders, tumours from patients treated with immunotherapy were characterized by increasing levels of CD15^+^ cancer cells (Fig. 5f and Extended Data Fig. 12b). Together, our findings show that the temporal trajectory of treatment effect is characterized by early infiltration of leukocytes, a reduction in cancer cells and a proportionate increase in stromal cells, but that this conserved pattern differs in degree according to response and treatment. We conclude that, despite a conserved pattern of treatment-induced cellular dynamics, immunotherapy distinctively remodels tumour structure.

## Dominant ICB response predictors

**Fig. 6:**
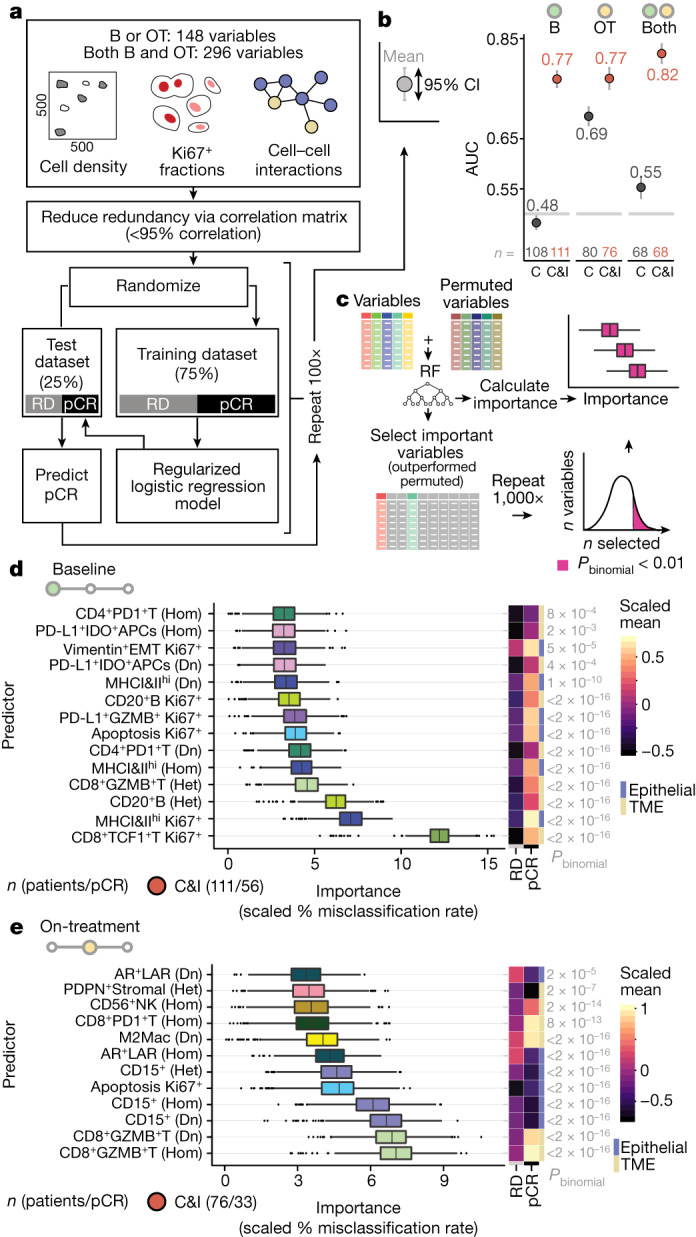
Multivariate modelling to predict ICB response. **a**, Analytical workflow for predictive modelling using multitiered multiplexed imaging data. Three models were trained using (1) baseline variables alone, (2) on-treatment variables alone or (3) combining baseline and on-treatment variables. **b**, AUC statistics for prediction probabilities derived from multivariate regularized logistic regression models to predict pCR. AUCs were computed using random held-out test data repeated 100 times, as described in **a**; circles are mean AUCs, error bars are 95% confidence intervals. **c**, Diagram illustrating variable importance analysis including all predictors described in **a**. **d**,**e**, Boxplots depicting baseline (**d**) and on-treatment (**e**) predictors of immunotherapy response (associated with a *P*_binomial_ < 0.01) ranked by importance in the model. On the right are heatmaps showing scaled mean values by response. *P*_binomial_ indicates *P* values derived from variable importance analysis illustrated in **c**. For all boxplots, boxes show 25th, 50th and 75th centiles; whiskers indicate 75th centile plus 1.5 × inter-quartile range and 25th centile less 1.5 × inter-quartile range; points beyond whiskers are outliers. Dn, density; Het, heterotypic interactions; Hom, homotypic interactions; RF, random forest.

In total, we derived 148 tissue features (densities of 37 cell phenotypes; 37 heterotypic, and 37 homotypic cell–cell interactions; 37 proliferative fractions) and found more were predictive of immunotherapy than chemotherapy response (112 versus 26), and more of these were found at baseline than on-treatment (70 versus 42). We therefore asked whether their combined predictive performance would also differ by treatment and timepoint. For each treatment arm, we fit three regularized multivariate logistic regression models: using baseline data, on-treatment data and data from both timepoints (Fig. 6a). Predictive performance was always better among immunotherapy-treated patients, implying that immunotherapy was more dependent on TME activation state and tumour structure (Fig. 6b). Despite finding many more predictors of immunotherapy response in treatment-naive baseline samples, predictive performance was similar for baseline and on-treatment multivariate models (mean receiver-operating characteristic area under the curve (AUC) 0.77 for both). Combining baseline and on-treatment features, however, materially improved predictive performance (mean AUC 0.82), showing that the features measured at each timepoint reflect distinct facets of response dynamics. Overall, multivariate modelling showed that TME activation and tumour structure play a greater role in treatment response when patients receive immunotherapy, and that early on-treatment biopsies improve predictive accuracy and could therefore help guide adaptive treatment strategies.

It remained unclear whether response to immunotherapy was driven by the combined effect of many features or just a few. We used an established method^20^ to identify the dominant drivers of response (Fig. 6c). This analysis revealed clear immunotherapy response drivers. At baseline, a total of 14 predictors contributed substantively to overall model performance, spanning cancer and TME cells, and mainly comprised cell–cell interactions and proliferative fractions (Fig. 6d). By far the best predictor was the proliferative fraction of CD8^+^TCF1^+^T cells, followed by the proliferative fraction of MHCI&II^hi^ cancer cells, cancer–B cell interactions and cancer–CD8^+^GZMB cell interactions. On-treatment, 12 top predictors were identified (Fig. 6e). The two best predictors of these 12 corresponded to CD8^+^GZMB^+^T cell abundance (homotypic interactions and density), whereas the next two most important predictors corresponded to CD15^+^ cancer cell abundance (homotypic interactions and density). In summary, feature importance analysis revealed distinct immunotherapy response drivers at baseline versus on-treatment.

## Discussion

By mapping the multicellular tumour ecosystem in situ in TNBC, we uncovered key ICB response predictors and showed that ICB distinctively remodels tumour structure.

The top two predictors of ICB response at baseline were the proliferative fractions of MHCI&II^hi^ cancer cells and CD8^+^TCF1^+^T cells. Past work has linked expression of MHCII by cancer cells to neoadjuvant ICB response in TNBC^21^. The triggers of aberrant cancer cell MHCII expression remain obscure but include local inflammatory signals such as interferon-γ (IFN-γ) and nuclear factor κB (NF-κB)^22^. Quiescent cancer cells residing in immunosuppressive niches in TNBC have been shown to resist ICB^23^ and could explain why lower MHCI&II^hi^ proliferative fractions are associated with ICB resistance. Proliferating CD8^+^TCF1^+^T cells, however, proved the dominant response driver. CD8^+^TCF1^+^T cells are thought to be a progenitor stem-like population that underlies the proliferative burst of CD8^+^ T cells after ICB^16^, predicts ICB response in melanoma^24^ and resides in intratumoural niches characterized by dense APCs^19^. Our finding that proliferating CD8^+^TCF1^+^T cells are more often in contact with MHCII^+^ cells supports the idea of an intratumoural niche, and links this population to MHCII expression. Proliferative CD8^+^ T cells positive for exhaustion markers have previously been characterized as a differentiating population that drives ICB response in melanoma^25^ and co-locates with T_reg_ cells in multicellular structures in breast tumours^13^. In addition to their heightened activation state and distinct spatial location, therefore, recent studies also corroborate proliferative CD8^+^TCF1^+^T cells as drivers of ICB response.

Proliferative fractions and cell–cell interactions were closely linked in our analysis and enriched for cells in distinct activation states. Past analyses of TNBC showed that the interface between cancer cells and infiltrating leukocytes defines diverse, spatially distinct TMEs^26,27^. Correlations with gene expression and markers of immune activity revealed that these diverse TMEs are also functionally distinct^26^. The intimate relationship between structure and function is supported by our findings that T cells contacting cancer cells are more often proliferative and express higher levels of activation markers. T cells reactive to neoepitopes are also characterized by distinct activation programmes that include expression of checkpoints such as PD-1 (refs. ^28,29^), raising the possibility that spatial cell–cell interactions enrich for these tumour-reactive T cells. The predictive power of cell–cell interactions may therefore be explained by their close alignment with the underlying function of both the participant cells and the wider immune response.

We found that on-treatment features improved predictive performance and that ICB induced distinctive changes on-treatment. In TNBC, past work showed that ICB induces clonal expansion of CD8^+^ T cells characterized by high *PRF1* and *GZMB* expression^30^. This suggests that on-treatment CD8^+^GZMB^+^T cell expansion is driven by ICB and explains why it proved the top on-treatment response predictor in our study. We also found that CD15^+^ cancer cells on-treatment predicted resistance to ICB. CD15^+^ breast cancer cells have been previously described^13,31^. CD15 is a carbohydrate blood group antigen, expressed by neutrophils and monocytes, that plays a role in cell adhesion^31^. In some instances (in keeping with previous findings^31^) we saw a striking mosaic expression pattern of intimately admixed CD15^+^ and CD15^−^ cancer cells, and heterotypic aggregations comprising CD15^+^ cancer cells surrounded by CD15^+^ leukocytes including neutrophils (Fig. 4f,g). Although the basis of our observation that on-treatment CD15^+^ cancer cells resist ICB is unclear, it supports the idea of discrete immunotherapy-resistant cell states in characteristic spatial contexts^23^. Finally, in addition to revealing markers of ICB effect, analysis of on-treatment samples also improved outcome prediction, highlighting the value of longitudinal sampling in neoadjuvant studies^32^.

In conclusion, we used IMC to precisely map the multicellular dynamics of ICB-treated TNBC. We found that key proliferative fractions and cell–cell interactions drive response, and that immunotherapy distinctively remodels tumour structure. Our findings indicate that cell phenotype, activation state and spatial organization collectively determine ICB effect. Systematic mapping of the intact tumour ecosystem could therefore enable precision immuno-oncology.

## Methods

### Study design and prospective tissue collection

Breast tumour samples were obtained from patients enrolled in a multicentre, randomized, open-label, phase III clinical trial^14^ (NeoTRIPaPDL1 or NeoTRIP; NCT02620280). NeoTRIP was a neoadjuvant immunotherapy trial of early high-risk TNBC in which 280 patients were randomized to receive neoadjuvant carboplatin and nab-paclitaxel on days 1 and 8, with or without atezolizumab (anti-PD-L1) on day 1 (Supplementary Tables 1–3). This treatment was given every 3 weeks (one cycle), for a total of eight cycles. Tumours were subsequently surgically excised and, if the responsible clinician opted to do so, an additional four cycles of anthracyclines were given. Patients with treatment-naive, early high-risk TNBC were eligible. Tumour receptor and PD-L1 status (by SP142, Ventana Medical Systems) were determined by central pathology review. Tumour infiltrating lymphocytes were also assessed using established methods at central pathology review^33^. The study protocol was approved at each participating institution; all patients provided written, informed consent.

Core biopsies for research were obtained at baseline and after one cycle of therapy (first day of second treatment cycle; on-treatment). Following the full course of therapy, tumours were surgically removed (post-treatment). Tissue microarrays (TMAs) were constructed for surgical excisions only. Regions of tumour, tumour–TME interface and adjacent stroma were annotated by a breast pathologist (G.V.) on corresponding H&E slides to guide TMA construction. Cores of 1 mm in diameter in the identified regions were removed and processed as TMAs.

### RNA sequencing and tumour molecular subtyping

Gene expression data were generated for the biopsy and surgical specimens using exome capture-based RNA sequencing on total RNA samples derived from 5 µm tumour sections. Briefly, exome-enriched complementary DNA libraries were constructed according to the manufacturer’s instructions (TruSeq RNA Exome, Illumina). Pools of 48 libraries were sequenced on a NextSeq 500 or NextSeq 2000 sequencing system (Illumina) with a high-output reagent kit for 75 base pair paired-end reads, with a mean of 10 million paired-end reads per sample per run. Pools were sequenced across replicate runs to achieve over 40 million paired-end reads per sample. Base call files from each sequencing run were converted to fastq format using bcl2fastq conversion software v.2.20, replicate fastq files for each sample were merged and files were aligned to the Ensembl GRCh37 *Homo sapiens* reference using STAR v.2.5.2 (ref. ^34^). Transcript assembly and expression analysis were performed on each sample with cufflinks v.2.2.1 (ref. ^35^), resulting in fragments per kilobase million (FPKM) values for each transcript in the genes of interest. The TNBC subtypes were determined using the minimal 101-gene TNBCtype^36^ (Supplementary Table 13). Briefly, gene expression for each sample for 101 genes was extracted from the whole transcriptome data and compared with five centroids representing each of the five subtypes by Pearson correlation. The sample was assigned to the subtype with the highest correlation. If no correlation was above 0.195, the subtype was not determined.

### Multiplexed imaging antibody panel

Candidate commercial antibodies intended for use in IMC were first validated by immunofluorescence using tonsil and breast cancer tissue to confirm optimal staining intensity, specificity and signal-to-noise ratio. Antibodies that passed validation by immunofluorescence were conjugated to metal isotopes and validated using IMC to ensure preservation of staining specificity and intensity. Sensitivity and specificity were further validated in multiplexed IMC experiments to ensure appropriate patterns of marker co-localization. Finally, optimal concentrations of all metal-conjugated antibodies were determined by visual inspection of IMC images in both tonsil and breast cancer tissue.

### Antibody conjugation

Indium, yttrium and lanthanide metals were conjugated to antibodies according to the manufacturer’s instructions (Maxpar X8 Multi-Metal Antibody Labelling Kit, Fluidigm). Platinum isotopes were conjugated directly to the reduced antibody without the polymer^37^. Conjugation of bismuth to the antibody required substitution of the L buffer from the Maxpar X8 labelling kit with 5% nitric acid (HNO_3_) during loading of the metal onto the polymer and deionized water (MilliQ) during the washes^38,39^. Metal-tagged antibodies were stored in a Candor Antibody Stabilizer (Candor Biosciences) at 4 °C. For a full list of antibodies and the metal conjugates, see Supplementary Table 6.

### Tissue labelling

FFPE slides were dewaxed in xylene and rehydrated in an alcohol gradient^12,40^. Tissue underwent antigen retrieval (Tris pH 9.0, 95 °C for 30 min) before blocking with 3% BSA in TBS for 1 h. Slides were incubated with unconjugated primary antibodies (PD-L1 clone SP142, PD-1 clone NAT105) overnight at 4 °C, then with metal-conjugated secondary anti-mouse and anti-rabbit antibodies for 3 h at room temperature. Next, slides were incubated with the remainder of the metal-tagged antibodies overnight at 4 °C, then with 0.5 μM iridium for DNA detection (Fluidigm, 201192B) for 30 min. Slides were washed with TBS 0.1% Tween between each labelling step, and air-dried following the final incubation.

### ROIs and IMC

Two sequential sections of FFPE tissue were prepared from core biopsy (baseline and on-treatment) and TMA blocks (post-treatment). One was stained with H&E using an autostainer (Leica ST5020 Stainer/TS5025 Transfer Station/CV5030 Coverslipper Workstation). H&E slides were scanned using the Leica Aperio AT2 Automated Digital Whole Slide Scanner. For core biopsies, ROIs measuring 500 × 500 µm^2^ were identified by a breast pathologist (H.R.A.) for acquisition by IMC using the Aperio eSlideManager web application (Leica Biosystems). Three ROIs were selected for each sample unless the biopsy was too small or, for baseline samples, contained no tumour cells. In on-treatment biopsies, if no invasive cancer was identified, regions of tumour bed were instead targeted for IMC.

The adjacent section was labelled with antibodies for IMC as described above. ROIs were mapped by manual inspection of annotated H&E images and subjected to IMC (Fluidigm): tissue was raster laser-ablated at 1 µm resolution, then ablated tissue aerosol was ionized using inductively coupled plasma, and resulting isotopic ion reporters quantified using time-of-flight mass spectrometry to infer protein abundance^3^.

### Spillover compensation

In mass cytometry applications such as IMC, signal from one channel may spill over to another channel due to trace amounts of contaminating isotopes in metal stock solutions. To account for this, all metal-conjugated antibodies in our panel were spotted separately onto glass slides and dried. Quantification by IMC of all metal isotopes in the panel was then conducted for each dried antibody spot on the slide. A ‘spillover matrix’ quantifying crosstalk was generated using the Bioconductor CATALYST^41^ package and subsequently used to correct single-cell measurements.

### Image processing, epithelial masks and single-cell measurements

This process is described by Extended Data Fig. 4a. Raw txt file data were converted into multistack image tiff files using existing software^42^. To identify regions of contiguous epithelium, we labelled pixels as epithelial based on their expression of cytokeratins and used a random-forest pixel classifier (Ilastik^43^) to assign all remaining pixels a probability of belonging to an epithelial region. Probability maps were saved as a red, green and blue (RGB) tiff file, and epithelial regions segmented to generate image masks using standard segmentation tools.

For segmentation of single cells, ‘salt and pepper’ noise was removed using a median filter and relevant channels rescaled per image to lie between zero and one. A two-channel image was passed to the Mesmer deep learning single-cell segmentation model^15^: a nuclear channel (sum of Histone H3 and Ir191) and a cytoplasmic channel (sum of panCK and CK5). Whole-cell image masks were used for downstream measurements (single-cell proteomic profiles and size) using CellProfiler^44^. Nuclei were mapped to whole-cell regions, and whole cells mapped to epithelial masks. To be considered ‘related’ to an epithelial mask, at least 30% of the pixels from a whole cell had to overlap with the epithelial region. Before taking measurements, multistack tiff files were filtered for single hot pixels. Single-cell proteomic measurements were taken by computing the mean ion count for each segmented whole cell; these data were spillover corrected using the spillover matrix described above with a non-negative least squares linear model implemented in CATALYST^41^. Small objects (cells with area less than 31 µm^2^) were excluded from analyses. We left one platinum isotopic channel empty for detection of carboplatin. Carboplatin signal detected in other platinum isotopes, for which conjugated antibodies were included (Vimentin and Calponin), was corrected by fitting a linear model to all cells: log-transformed cell expression was predicted using the carboplatin isotope, and the resulting model residuals were taken as corrected values.

### Image curation and cell phenotyping

Cell phenotypes were assigned by semi-supervised clustering. Cells were first classified as epithelial or TME using multiple classification methods, in which the best performing method for each image was manually selected by visual inspection of tissue morphology and cytokeratin expression (Extended Data Fig. 4a,b). The classification methods are listed below.

A two-component Gaussian mixture model was fit to the log-transformed sum of all cytokeratins (panCK, CK8/18, CK5/14) to distinguish cells as positive or negative for cytokeratin. Cells related to epithelial masks based on a 30% area overlap were deemed ‘mask-positive’. All images were annotated with mask-positive, cytokeratin-positive or double-positive cells (those that were positive by both criteria). All images were inspected, using tissue morphology as the standard, to determine which method best captured epithelial cells. Most epithelial cells were accurately classified by this approach, but some infiltrating leukocytes were misclassified. To capture infiltrating leukocytes, cells that were mask-positive and cells that were cytokeratin-positive were subclustered using a combination of key epithelial (panCK, CK8/18, CK5/14, AR, GATA3) and immune markers (CD3, CD4, CD8, CD68, CD163, CD11c). Inspection of average expression profiles per cluster was used to identify infiltrating leukocytes. This process was repeated until satisfactory results (determined by inspection of images annotated with cell phenotypes) were obtained. Tumours poorly classified by these approaches (often owing to low cytokeratin expression) were subjected to unsupervised clustering by Phenograph^45^ per image. Every marker (except for DNA, H3, Carboplatin, c-PARP, CD68, Calponin and Caveolin) was used for clustering, and values were rescaled to lie between zero and one per image. Clusters were categorized as either epithelial or TME by manual inspection of annotated images.

Cell phenotypes were derived separately for epithelial and TME cells. Only proteins known to be expressed by epithelial or TME cells based on previous knowledge or manual inspection were included. The proliferation marker Ki67 was excluded from cell clustering. Expression values were clipped at the 99th centile, mean centred and scaled before clustering. Clustering was performed in two steps. First, a self-organizing map (SOM) was created using GigaSOM^46^, then median expression values per SOM node were passed to Phenograph^45^ and resulting clusters mapped back to single cells. Heatmaps of scaled median expression values were inspected, and clusters lacking meaningful differences merged. Resulting clusters were labelled based on their expression profiles. Cluster validity was further investigated by inspecting images annotated with cluster labels and expression profiles to ensure cell morphology and expression values were concordant with the cluster label.

Extensive image curation was conducted under the supervision of a pathologist (H.R.A.) to identify invasive cancer cells and exclude in situ or normal epithelial cells from clinical correlative analyses. All TME cells were retained for downstream analysis. For a full breakdown of cell numbers and image numbers acquired for this study, refer to Supplementary Tables 4 and 5.

### Thresholds for marker positivity

We identified thresholds for assigning a cell as ‘positive’ for a given marker by inspecting a random selection of at least 50 images for which cells passing a quantile threshold (calculated using all data) were highlighted. This procedure was repeated at differing quantile thresholds until the value that most closely aligned with marker positivity was identified.

### Differential cell phenotype abundance analysis

We used generalized linear models under a binomial distribution with logit link function to determine whether abundance of a given cell phenotype differed between categorical groups^47^ (PD-L1 status and tumour transcriptional subtype). Cell phenotype proportion per tumour was taken as the response variable and predicted by the binary predictor. The precision of proportion estimates varied substantially between tumours because the total number of cells sampled was also highly variable. When more cells were sampled, proportion estimates were more precise. To account for this variable precision, generalized linear models were weighted by the total number of cells. Proportion values were computed separately by epithelial or TME compartments. The same approach was taken to investigate the relationship between stromal infiltrating lymphocytes and cell phenotype proportions, fitting stromal infiltrating lymphocytes as a continuous predictor. Model coefficients were exponentiated, log_2_-transformed and reported as log_2_ odds ratios. *P* values were adjusted for multiple testing by the Benjamini–Hochberg method.

### Cell densities

Cell phenotype densities were calculated by dividing the number of total cells obtained per biopsy by the total area of the tissue acquired (per mm^2^; Fig. 2a). As tissue did not cover the entirety of all ROIs, the convex hull method was used to draw a ‘tissue’ area based on the existence of all segmented cells. Summary values, *P* values and point estimates for associations between cell densities and response can be found in Supplementary Tables 7 and 8.

### Cell–cell interaction metrics

Cells were defined as participating in an interaction if their whole-cell masks were in direct contact (that is, their pixels were contiguous; Fig. 2e). Taking direct contact as the criterion, we computed interactions for all cells using CellProfiler. Cell phenotypes were mapped to cell–cell interaction maps. For each tumour, four ‘flavours’ of cell–cell interaction metric were computed (epithelial homotypic (all epithelial cells interacting with each of the 17 epithelial phenotypes), epithelial heterotypic (all epithelial cells interacting with each of the 20 TME phenotypes), TME homotypic (all TME cells interacting with each of the 20 TME phenotypes) and TME heterotypic (all TME cells interacting with each of the 17 epithelial phenotypes)). The homotypic interactions for an epithelial cell phenotype of interest, for example, were computed as the total number of interactions between that phenotype and all other epithelial cells (regardless of phenotype), divided by the total number of cells in the tumour sample (epithelial and TME cells combined). By contrast, the epithelial heterotypic interactions for a TME phenotype of interest were computed as the number of epithelial–TME interactions (with that TME phenotype) divided by the total number of cells. The definitions for TME-centric interactions were the same but computed from the perspective of the TME (heterotypic interactions were with different epithelial cell phenotypes). Summary values, *P* values and point estimates for associations between cell–cell interactions and response can be found in Supplementary Tables 9 and 10.

### Proliferative fractions

The proportion of cells positive for the proliferation marker Ki67 was computed per cell phenotype per tumour per timepoint (Fig. 3a). When a cell phenotype was absent, its corresponding proliferative fraction was also zero. Ki67 status per cell was determined using the method described above to find a suitable threshold for positivity. Summary values, *P* values and point estimates for associations between proliferative fractions and response can be found in Supplementary Table 11.

### Associations with immunotherapy response

We used pCR as a response end point and fitted univariate logistic regression models to test for associations between response and tissue features. Plots illustrating estimates of association between tissue features and pCR depict two odds ratios (and 95% confidence intervals) per predictor: one for each treatment arm, resulting from a univariate logistic regression model restricted to the relevant (C or C&I) study population. Adjacent to these two odds ratios, *P* values for statistical interaction (*P*_interaction_) are depicted: these were derived from trivariate logistic regression models that included the feature of interest, treatment (C or C&I) and a term for statistical interaction between the feature of interest and treatment. *P*_interaction_ values corresponded to the statistical term for interaction computed in these models. All predictors (cell densities, cell–cell interactions and proliferative fractions) were square-root-transformed and modelled as continuous. Model coefficients and 95% confidence intervals were exponentiated and reported as odds ratios. When necessary, predictor values were multiplied (proliferative fractions by 10, and cell–cell interactions by 100) so that more interpretable odds ratios could be derived. All clinical correlative analyses were limited to the per-protocol patient population (*n* = 258; that is, patients who were treated according to the entire trial protocol). To account for multiple testing and to evaluate the likelihood of false positives among significant associations, we computed the FDR using the Benjamini–Hochberg method.

### Differential T cell activation

To compare the differential activation state between T cells in contact with tumour cells versus those not in contact, we computed the mean expression level (of activation markers TOX, PD-1, GZMB, OX40, ICOS) per tumour per timepoint for each group of T cells (in contact and not) and compared the resulting distributions using two-sided Wilcoxon tests. The same method, taking per-tumour averages, was deployed for comparison of the proportion of Ki67^+^ cells. The same analysis was conducted when comparing proliferating versus non-proliferating CD8^+^TCF1^+^T cells.

### Immunotherapy-induced tissue dynamics

We compared the cellular composition of tumours through treatment to identify changes that characterized sensitivity and resistance to immunotherapy. We plotted the mean proportion of each cell phenotype (computed separately for epithelial and TME compartments) across timepoints, treatments and response. Means were *Z*-scored per phenotype and illustrated as trend plots. Significant differences between treatments were illustrated as boxplots and tested using a two-sided Wilcoxon test.

### Multivariate modelling and variable importance

We fitted regularized logistic regression models (using the R package glmnet) to determine the discriminatory performance of tissue features taken in aggregate to predict pCR. We derived three distinct sets of features for each tumour, separating epithelial and TME compartments: cell phenotype densities; cell interaction metrics as described above; proliferative fractions of cell phenotypes.

A total of 148 variables were derived for single timepoints and 296 when baseline and on-treatment timepoints were combined. Only variables with more than six unique values across samples were retained. We further reduced the feature space for multivariate models by identifying groups of highly correlated variables (Spearman rank correlation > 0.95) and selecting one representative variable at random. To identify highly correlated groups, we first built a graph of variables with at least one correlation of greater than 0.95 (edges were weighted by the correlation coefficient) and used Louvain clustering to discretise subgraphs representing groups of highly correlated variables. Next, data were randomly split into training (75%) and test (25%) sets, for which the proportion of responders was approximately balanced between the two. Regularized logistic regression models were fitted to the training set (using cross validation to identify the minimal shrinkage factor lambda) and predictions made using the test data. An AUC statistic was computed using the prediction probabilities in the test data. To estimate the precision of AUC values, and to derive 95% confidence intervals, the random split procedure, model fitting and testing were repeated 100 times. This whole process was conducted separately by treatment arm, by timepoint (baseline and on-treatment) and for a combined model (data from both baseline and on-treatment timepoints).

To determine which predictors were most important in driving predictions we used an established feature selection algorithm (implemented in the R package Boruta)^20^. The principle of this method is repeated comparison of true values with randomly shuffled features to identify which outperform random data more often than would occur by chance. Briefly, all predictors are randomly shuffled, doubling the original predictor set (the original plus the shuffled data), and a random-forest classifier fitted to determine the importance of all predictors in the doubled dataset (importance is calculated by replacing a feature with its randomly permuted equivalent and computing the resulting percentage of misclassified tumours, and then scaled by dividing by the standard error from all misclassification rates). The maximum feature importance achieved among all the randomly shuffled predictors is the threshold for a true feature to then be deemed important (on the basis that an important predictor must outperform random equivalents). This process was repeated 1,000 times to generate a binomial distribution of the number of times a given feature was regarded as important, and a final set of important variables identified based on a threshold of *P* < 0.01. Taking only those features that outperformed randomly shuffled data more often than expected by chance (at Bonferroni-corrected *P* < 0.01), we plotted the distribution of their importance values (the scaled percentage misclassification rate) across all 1,000 runs to rank their importance.

### Reporting summary

Further information on research design is available in the Nature Portfolio Reporting Summary linked to this article.

## Online content

Any methods, additional references, Nature Portfolio reporting summaries, source data, extended data, supplementary information, acknowledgements, peer review information; details of author contributions and competing interests; and statements of data and code availability are available at 10.1038/s41586-023-06498-3.

### Supplementary information

Supplementary Information Supplementary Figs. 1 and 2 and legends for Supplementary Tables.

Reporting Summary

Supplementary Tables Supplementary Tables 1–13.

## Data Availability

All imaging mass cytometry and clinical response data can be accessed via a Zenodo data repository (10.5281/zenodo.7990870) for academic non-commercial research. For commercial access, parties will be directed to an appropriate contact. Gene expression data used to call transcriptomic subtypes of TNBC together with subtype assignments are provided in Supplementary Table 13.

## Code availability

Code for image processing is hosted at https://github.com/BodenmillerGroup/ImcSegmentationPipeline and https://github.com/vanvalenlab/deepcell-tf. All additional analysis code can be accessed via 10.5281/zenodo.7990870 alongside study data.
