# Supplementary material for: Spatial predictors of immunotherapy response in triple-negative breast cancer
Source: Nature. 2023 Sep 6;621(7980):868–76. doi: 10.1038/s41586-023-06498-3 (PMC10533410; doi:10.1038/s41586-023-06498-3)
Supplement: Supplementary file 1 — Supplementary Figs. 1 and 2 and legends for Supplementary Tables. [file 41586_2023_6498_MOESM1_ESM.pdf]

nature portfolio

https://doi.org/10.1038/s41586-023-06498-3

Supplementary information

Spatial predictors of immunotherapy response in triple-negative breast cancer

In the format provided by the authors and unedited

Annotation for supplementary material associated with *Spatial predictors of immunotherapy response in triple negative breast cancer*

Xiao Qian Wang[1], Esther Danenberg[1], Chiun-Sheng Huang[2], Daniel Egle[3], Maurizio Callari[4], Begoña Bermejo[5,6,7], Matteo Dugo[8], Claudio Zamagni[9], Marc Thill[10], Anton Anton[11], Stefania Zambelli[8], Stefania Russo[12], Eva Maria Ciruelos[13], Richard Greil[14,15,16], Balázs Győrffy[17,18], Vladimir Semiglazov[19], Marco Colleoni[20], Catherine M. Kelly[21], Gabriella Mariani[22], Lucia Del Mastro[23,24], Olivia Biasi[20], Robert S. Seitz[25], Pinuccia Valagussa[4], Giuseppe Viale[20,26], Luca Gianni[4,28], Giampaolo Bianchini[4,8,28*], H. Raza Ali[1,27,28*]

[1] CRUK Cambridge Institute, University of Cambridge, Cambridge, UK
[2] National Taiwan University Hospital, College of Medicine, National Taiwan University and Taiwan Breast Cancer Consortium, Taipei, Taiwan
[3] Department of Gynecology, Brust Gesundheit Zentrum Tirol, Medical University Innsbruck, Innsbruck, Austria
[4] Fondazione Michelangelo, Milan, Italy
[5] Medical Oncology, Hospital Clínico Universitario de Valencia, Biomedical Research Institute INCLIVA, Valencia
[6] Medicine Department, Universidad de Valencia.
[7] Oncology Biomedical Research National Network (CIBERONC-ISCIII), Madrid.
[8] San Raffaele Hospital, Milano, Italy
[9] IRCCS Azienda Ospedaliero-universitaria di Bologna, Italy
[10] Department of Gynecology and Gynecological Oncology, Agaplesion Markus Krankenhaus, Frankfurt am Main, Germany
[11] Hospital Universitario Miguel Servet, Zaragoza, Spain
[12] Department of Oncology, Azienda Sanitaria Universitaria Friuli Centrale, Udine, Italy
[13] Hospital Universitario 12 de Octubre, Madrid, Spain
[14] 3rd Medical Department, Paracelsus Medical University Salzburg, Salzburg, Austria
[15] Salzburg Cancer Research Institute-CCCIT, Salzburg, Austria
[16] Cancer Cluster Salzburg, Salzburg, Austria
[17] Semmelweis University Dept. of Bioinformatics, Budapest, Hungary
[18] Cancer Biomarker Research Group, Research Centre for Natural Sciences, Institute of Enzymology, Budapest, Hungary
[19] NN Petrov Research Institute of Oncology, St. Petersburg, Russia
[20] IEO, Istituto Europeo di Oncologia, IRCCS, Milan, Italy
[21] Mater Private Hospital, Dublin and Cancer Trials Ireland Breast Group
[22] Fondazione IRCSS - Istituto Nazionale Tumori, Milan, Italy
[23] IRCCS Ospedale Policlinico San Martino, UO Clinica di Oncologia Medica, Genoa, Italy
[24] Università di Genova, Dipartimento di Medicina Interna e Specialità Mediche (Di.M.I.), Genoa, Italy
[25] Oncocyte Corporation, Irvine, California, USA
[26] University of Milan, Milan, Italy
[27] Department of Histopathology, Addenbrookes Hospital, Cambridge, UK
[28] These authors jointly supervised this work
*Correspondence: bianchini.giampaolo@hsr.it ; raza.ali@cruk.cam.ac.uk

---

Table of Contents:

Supplementary Figures:

- **Supplementary Fig. 1|** Phenotypic composition of TNBC during neoadjuvant therapy (pg2)
- **Supplementary Fig. 2 |** Cell counts per biopsy grouped by proliferative fraction quantiles (pg3)

Supplementary Tables:

Table	Description
Table 1	Patient demographics and clinical characteristics - Please note that this is data for all 280 patients enrolled in the trial, of which 279 were analysed in this study.
Table 2	Patient exclusions, including reasons
Table 3	Paired counts - no. of samples available at multiple timepoints
Table 4	Counts used in most analyses - images containing invasive epithelial cells
Table 5	Counts by image type - no. of images containing only TME cells and number of images containing non-invasive epithelial cells
Table 6	List of ethics committees that approved of the NeoTRIPaPDL1 study
Table 7	Summary of cell phenotype densities across timepoints, treatment arms and response
Table 8	Point estimates, confidence intervals, false discovery rates and p-values of associations between cell densities and response
Table 9	Summary of cell-cell interaction values across timepoints, treatment arms and response
Table 10	Point estimates, confidence intervals, false discovery rates and p-values of associations between cell-cell interactions and response
Table 11	Point estimates, confidence intervals, false discovery rates and p-values of associations between proliferation fraction and response
Table 12	List of antibodies and metal conjugates in the panel

Abbreviations	Full
TME	Tumour microenvironment
B	Baseline
OT	On-treatment
PT	Post-treatment
C	Chemotherapy
C&I	Chemotherapy and Immunotherapy
RD	residual disease
pCR	pathological complete response
fdr	false discovery rate
estimate	point estimate derived from logstic regression
std.error	standard error
conf.low	lower bound of 95% confidence interval
conf.high	upper bound of 95% confidence interval

Each supplementary table is on its own sheet in the excel spreadsheet.

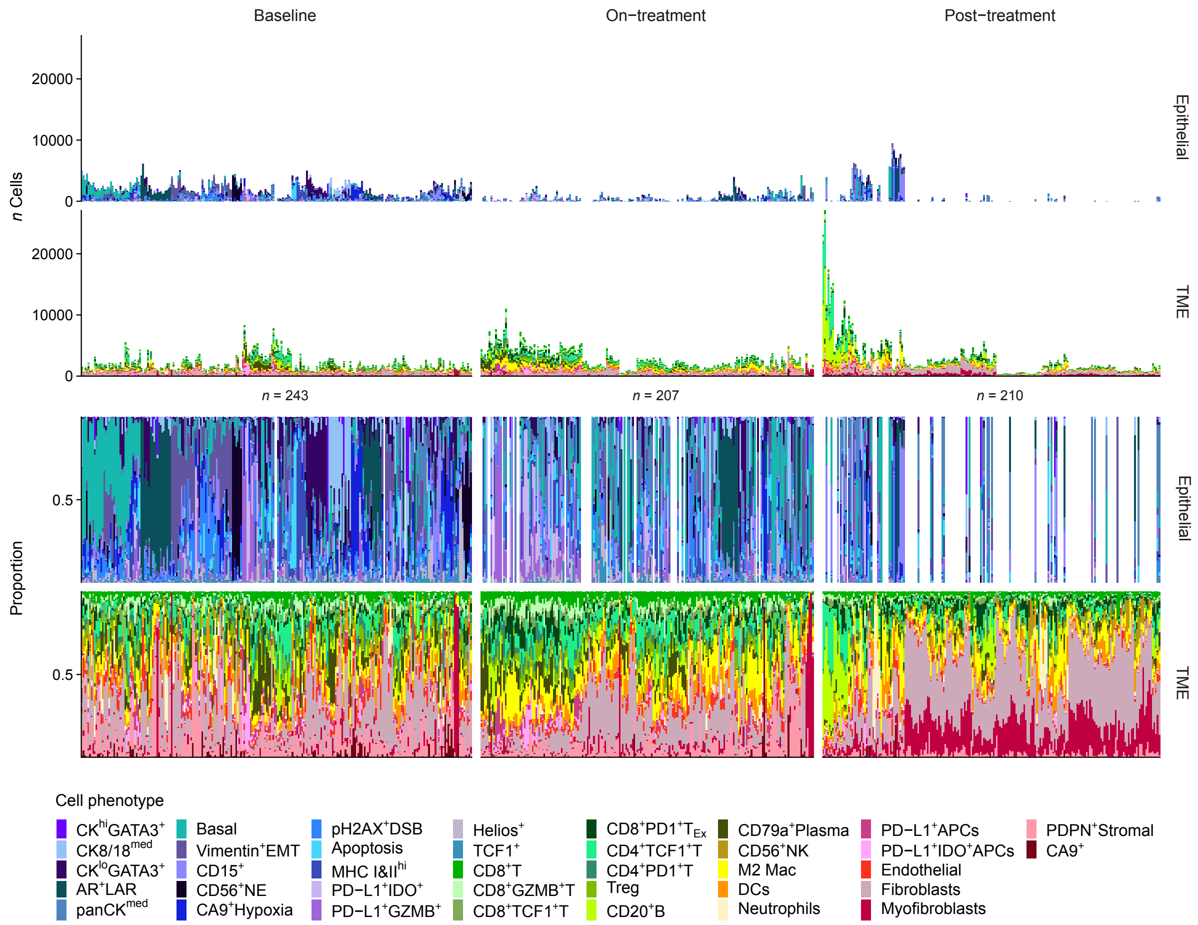

Supplementary Fig. 1| Phenotypic composition of TNBC during neoadjuvant therapy. Number and proportion of cell phenotypes by compartment (epithelial or TME) are depicted for each patient at each timepoint.

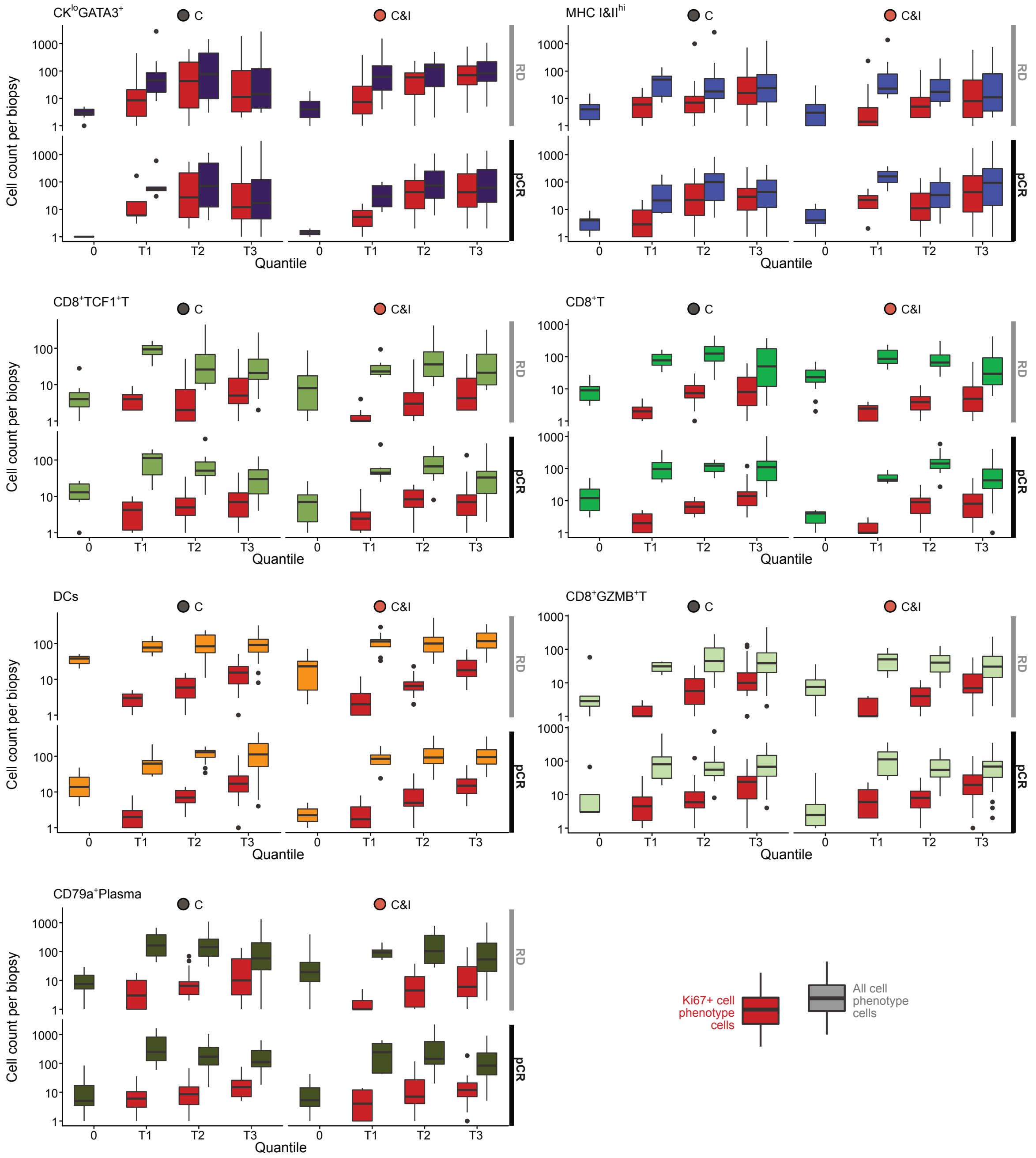

Supplementary Fig. 2 | Cell counts per biopsy grouped by proliferative fraction quantiles. Boxplots depict comparisons between absolute number of Ki67+ cells of specific phenotypes (red) next to number of total cells of specific phenotypes per biopsy per quantile shown in Fig. 3D. Boxes show 25th, 50th, and 75th centiles; whiskers indicate 75th centile plus 1.5x inter-quartile range and 25th centile less 1.5x inter-quartile range; points beyond whiskers are outliers. Numbers of patients for each group are the same as represented in Fig. 3D.
