# Supplementary material for: Spatial predictors of immunotherapy response in triple-negative breast cancer
Source: Nature. 2023 Sep 6;621(7980):868–76. doi: 10.1038/s41586-023-06498-3 (PMC10533410; doi:10.1038/s41586-023-06498-3)
Supplement: Supplementary file 2 — Reporting Summary [file 41586_2023_6498_MOESM2_ESM.pdf]

nature portfolio

Corresponding author(s): Giampaolo Bianchini, H Raza Ali

Last updated by author(s): May 22, 2022

Reporting Summary

Nature Portfolio wishes to improve the reproducibility of the work that we publish. This form provides structure for consistency and transparency in reporting. For further information on Nature Portfolio policies, see our Editorial Policies and the Editorial Policy Checklist.

Statistics

For all statistical analyses, confirm that the following items are present in the figure legend, table legend, main text, or Methods section.

n/a	Confirmed	
☐	☒	The exact sample size (*n*) for each experimental group/condition, given as a discrete number and unit of measurement
☐	☒	A statement on whether measurements were taken from distinct samples or whether the same sample was measured repeatedly
☐	☒	The statistical test(s) used AND whether they are one- or two-sided<br>*Only common tests should be described solely by name; describe more complex techniques in the Methods section.*
☐	☒	A description of all covariates tested
☐	☒	A description of any assumptions or corrections, such as tests of normality and adjustment for multiple comparisons
☐	☒	A full description of the statistical parameters including central tendency (e.g. means) or other basic estimates (e.g. regression coefficient) AND variation (e.g. standard deviation) or associated estimates of uncertainty (e.g. confidence intervals)
☐	☒	For null hypothesis testing, the test statistic (e.g. F, t, r) with confidence intervals, effect sizes, degrees of freedom and P value noted<br>*Give P values as exact values whenever suitable.*
☒	☐	For Bayesian analysis, information on the choice of priors and Markov chain Monte Carlo settings
☒	☐	For hierarchical and complex designs, identification of the appropriate level for tests and full reporting of outcomes
☐	☒	Estimates of effect sizes (e.g. Cohen's d, Pearson's r), indicating how they were calculated

Our web collection on statistics for biologists contains articles on many of the points above.

Software and code

Policy information about availability of computer code

Data collection	Aperio eSlideManager (Leica Biosystems) - viewing H&E slides and annotating ROI selection<br>CYTOF Software v7.0 (Fluidigm) - for running of the Hyperion imaging mass cytometry system and acquisition of samples

April 2023

Data analysis

Image Processing:
>python v3.8 (to run imctools and DeepCell)
>imctools package v1.0.8 (https://github.com/BodenmillerGroup/imctools)- for conversion of .txt files to .tiff files
>DeepCell package v0.11.0 (https://github.com/vanvalenlab/deepcell-tf, Greenwald, N.F., Miller, G., Moen, E. et al. Whole-cell segmentation of tissue images with human-level performance using large-scale data annotation and deep learning. Nat Biotechnol 40, 555–565 (2022)) - for single-cell segmentation
>CellProfiler v4.0.6 (https://cellprofiler.org/) - generating single cell, epithelial and vessel masks
>Ilastik v1.3 - training epithelial/vessel masks

Cell Clustering:
>GigaSOM v0.6.6 (https://github.com/LCSB-BioCore/GigaSOM.jl)
>Phenograph v0.99.1(https://github.com/JinmiaoChenLab/Rphenograph)

Statistical Analysis:
R v3.5.1
A full list of packages with versions and details about the exact virtual environment used can be found in the conda environment provided with the data (condaEnv.yml) found here: https://doi.org/10.5281/zenodo.7990870

For manuscripts utilizing custom algorithms or software that are central to the research but not yet described in published literature, software must be made available to editors and reviewers. We strongly encourage code deposition in a community repository (e.g. GitHub). See the Nature Portfolio guidelines for submitting code & software for further information.

Data

Policy information about availability of data

All manuscripts must include a data availability statement. This statement should provide the following information, where applicable:

- Accession codes, unique identifiers, or web links for publicly available datasets
- A description of any restrictions on data availability
- For clinical datasets or third party data, please ensure that the statement adheres to our policy

All imaging mass cytometry and clinical response data have been placed in the public domain in a Zenodo data repository (https://doi.org/10.5281/zenodo.7990870) for academic non-commercial research. For commercial access parties will be directed to an appropriate contact. Gene expression data used to call transcriptomic subtypes of TNBC together with subtype assignments are provided in Supplementary Table 13.

Research involving human participants, their data, or biological material

Policy information about studies with human participants or human data. See also policy information about sex, gender (identity/presentation), and sexual orientation and race, ethnicity and racism.

Reporting on sex and gender

As part of the inclusion criteria, only female patients were eligible to participate in the NeoTRIP clinical trial.

Reporting on race, ethnicity, or other socially relevant groupings

Patients were recruited from multiple centres across Europe and Asia. Information about race and ethnicity of patients was not provided for this study.

Population characteristics

As part of the inclusion criteria, only patients over the age of 18 with early high-risk (T1cN1; T2N1; T3N0) or locally advanced and inflammatory ductal breast cancers (stage III A-C according to AJCC) suitable for neoadjuvant treatment were eligible to participate. For the 280 patients enrolled in the NeoTRIPaPDL1 trial, the age range was 24-79, with a median age of 50. Of these 280 enrolled patients, 279 were analysed in this study. Further details about the patients enrolled in the trial can be found in Supplementary Table 1.

Recruitment

Physicians recruited patients who attended participating clinical centres if they met the inclusion criteria (listed above) and consented to the study. Key exclusion criteria were metastatic disease (stage IV), bilateral breast cancer, other malignancies, inadequate bone marrow or renal function, impaired liver function, impaired cardiac function, uncontrolled hypertension, pregnancy, refusal to use contraception and history of autoimmune disease.

It is expected that the patients recruited would be representative of the overall population of patients who fit the inclusion criteria in routine clinical practice. There could be a bias of certain patients being more willing to consent to the study, but it is unknown how this would affect our analyses.

Ethics oversight

NeoTRIPaPDL1 trial is a multicentric international study involving 41 active centers from Italy, Spain, Russia, Germany, Austria, Ireland and Taiwan. First approval for the study protocol was obtained from the Independent Ethics Committee of the San Raffaele Scientific Institute in Milan. Following approvals for the study protocol (and any modifications thereof) were obtained from independent ethics committees at each participating site:

Comitato Etico dell'IRCCS Ospedale San Raffaele di Milano
Comitato Etico Unico Regionale
Comitato Etico Provinciale di Reggio Emilia
Comitato Etico Azienda Ospedaliero-Universitaria di Bologna
Comitato Etico della Fondazione IRCCS Istituto Nazionale dei Tumori, Milano
Comitato Etico Regionale della Liguria
Comitato Etico Interaziendale Milano Area A

Comitato Etico degli IRCCS Istituto Europeo di Oncologia e Centro Cardiologico Monzino
Comitato Etico Fondazione Piemonte per l'Oncologia
Comitato Etico Regionale Sezione Area Vasta Sud Est
Ethikkommission Land Salzburg
Ethikkommissionbei der LMU Muenchen
Clinical Research Ethics Committee University College Cork
Ethics Committee Federal State Budgetary Institution "N.N. Petrov Research Institute of Oncology" of the Ministry of Health of the Russian Federation
Ethics Committee of "Komanda" LLC
Ethics Committee of the N.N. Blokhin Russian Cancer Research Centre
Ethics Committee of the State Budgetary Institution of Healthcare "St. Petersburg Clinical Scientific Center of Specialized Services of the Medical Assistance (Oncology)"
Comité Etico de Investigacion Clinica del Hospital Clinico Universitario de Valencia
Research Ethics Committee D,
National Taiwan University Hospital
Institutional Review Board Committee A Chianghua Christian Hospital
Institutional Review Board Taipei Veterans General Hospital
Institutional Review Board Kaohsiung Medical University Chung-Ho Memorial Hospital
Research Ethics Committee China Medical University & Hospital

This information is included as Supplementary Table 6 along with a list of participating investigators and sites.

Note that full information on the approval of the study protocol must also be provided in the manuscript.

Field-specific reporting

Please select the one below that is the best fit for your research. If you are not sure, read the appropriate sections before making your selection.

☒ Life sciences ☐ Behavioural & social sciences ☐ Ecological, evolutionary & environmental sciences

For a reference copy of the document with all sections, see nature.com/documents/nr-reporting-summary-flat.pdf

Life sciences study design

All studies must disclose on these points even when the disclosure is negative.

Sample size	The sample size calculation for the NeoTRIPaPDL1 clinical trial is described in Gianni et al. 2022, Annals of Oncology. In sum, sample size was calculated based on the comparison of the primary parameter EFS between the two treatment groups, by a log-rank test in the context of a group-sequential design. Sample size was not calculated for correlative studies. Analysis was conducted using all available tissue.
Data exclusions	As detailed in Supplementary Table 2, some tumours were not assessed by IMC due to either having no tissue available for sampling, or no areas of invasive tumour to image. Similarly, epithelial cells in acquired images that were not invasive tumour cells were annotated and excluded from analysis. As each acquired image was manually checked, we excluded images that had poor data quality (very few cells, poor staining, bad tissue quality) as these were not representative of the TME. No other data points/outliers have been excluded in our subsequent analyses.
Replication	NeoTRIPaPDL1 is a randomised controlled trial involving human participants. Findings were not validated (replicated) in a second clinical trial.
Randomization	Patients were randomised computationally by geographical area, disease stage (early high-risk vs locally advanced) and PD-L1 expression (yes or no) as described in Gianni et al. 2022, Annals of Oncology.
Blinding	This study was open-label (both participants and investigators knew treatment assignment). The surrogate endpoint is pathological complete response and the final endpoint is event-free survival after 5 years. Researchers conducting correlative analyses were not blinded to clinical data.

Reporting for specific materials, systems and methods

We require information from authors about some types of materials, experimental systems and methods used in many studies. Here, indicate whether each material, system or method listed is relevant to your study. If you are not sure if a list item applies to your research, read the appropriate section before selecting a response.

Materials & experimental systems

n/a	Involved in the study	
☐	☒	Antibodies
☒	☐	Eukaryotic cell lines
☒	☐	Palaeontology and archaeology
☒	☐	Animals and other organisms
☐	☒	Clinical data
☒	☐	Dual use research of concern
☒	☐	Plants

Methods

n/a	Involved in the study	
☒	☐	ChIP-seq
☒	☐	Flow cytometry
☒	☐	MRI-based neuroimaging

Antibodies

Antibodies used

List all all antibodies used along with their staining concentrations can be found in Supplementary Table 6.

Validation

Antibody validation occurred in multiple stages. Candidate commercial antibodies intended for use in IMC were first validated by immunofluorescence (IF) using tonsil and breast cancer tissue to confirm optimal staining intensity, specificity, and signal-to-noise ratio. Antibodies that passed validation by IF were conjugated to metal isotopes and validated using IMC to ensure preservation of staining specificity and intensity. Sensitivity and specificity were further validated in multiplexed IMC experiments to ensure appropriate patterns of marker co-localisation. Finally, optimal concentrations of all metal-conjugated antibodies were determined by visual inspection of IMC images in both tonsil and breast cancer tissue following antibody titration. The final antibodies and their staining concentrations used for the study can be found in Supplementary Table 6.

Clinical data

Policy information about clinical studies

All manuscripts should comply with the ICMJE guidelines for publication of clinical research and a completed CONSORT checklist must be included with all submissions.

Clinical trial registration

NCT02620280

Study protocol

Clinical trial findings have been published:
Gianni, L., Huang, C. S., Egle, D., Bermejo, B., Zamagni, C., Thill, M., . . . Viale, G. (2022). Pathologic complete response (pCR) to neoadjuvant treatment with or without atezolizumab in triple-negative, early high-risk and locally advanced breast cancer: NeoTRIP Michelangelo randomized study. Annals of Oncology. doi:https://doi.org/10.1016/j.annonc.2022.02.004

Data collection

A full list of participating research groups, investigators and sites can be accessed at Gianni et al. 2020 Annals of Oncology. To summarise, patients were recruited by the Taiwan Breast Cancer Consortium, Cancer Trials Ireland and Michelangelo (Austria, Germany, Russia, Spain and Italy). The trial opened for recruitment on April 2016 and the last patient was randomised December 28, 2018. Final pCR assessment was completed September 2019. Biopsies and surgical excisions were sectioned from February-April 2021.

Outcomes

The primary outcome of the clinical trial is to compare event-free survival in the two treatment arms 5 years after randomisation of the last patient. However, correlative analyses utilising the clinical data was performed with the surrogate endpoint, pathological complete response (pCR), defined as the absence of invasive cells in the breast and lymph nodes. This was assessed by a pathologist on sections of the surgical excision.
